# Supplementary material for: Simplified sewerage to prevent urban leptospirosis transmission: a cluster non-randomised controlled trial protocol in disadvantaged urban communities of Salvador, Brazil
Source: BMJ Open. 2023 Jun 23;13(6):e065009. doi: 10.1136/bmjopen-2022-065009 (PMC10314607; doi:10.1136/bmjopen-2022-065009)

Supplementary material

Supplementary Table 1. Specific age range for each outcome.

Outcome	Age group
Leptospirosis, preventive practice, well-being, perceptions of residents	≥ 18 years
Arboviral diseases	≥ 6 months
Long-term infant development	≥ 6 to ≤ 66 months
Enteric diseases	≥ 6 to ≤ 66 months

Supplementary Table 2. Primary and secondary outcomes baseline in assessed in this study protocol, Salvador, Brazil, 2022

Outcomes of interest	Operational definition
**1. Individual *Leptospira* infection**	
	> *Leptospira* infection defined as a four-fold rise in microscopic agglutination test (MAT) titer or seroconversion (negative to ≥1:50) between samples from consecutive serosurveys.
**2. Individual risk for *Leptospira* exposure**	
	> Walk-in flooding in the neighborhood
	> Walk barefoot outside the household
	> Need to unclog sewerage
	> Perception of vulnerability to leptospirosis
	> Perception of the severity of the leptospirosis disease
	> Perception of the presence of rats near the house
**3. Environmental for *Leptospira* exposure**	
	> Proximity and contact frequency to open sewer, trash, and other exposures to environmental sources
	> Sewage infrastructure at home and in the community
	> Flooding in the community and inside the home
	> Floor and road conditions
	> Accumulation of garbage near the house
	> Presence and concentration of *Leptospira* load in the soil
**4. Sanitation-related knowledge and practice**	
	Consequences of lack or poor sanitation as:
	> Structural problems
	> Major related diseases
	> Institutions responsible for providing sanitation in the community
	> Practice related to sanitation in-home and community
**5. Social Capital**	
	> Self-Assessment of coexistence and trust with neighbors.
	> Perception of neighbors' willingness to help each other
	> Social, leisure, and community support activities practiced
	> Willingness to continue living in the community
**6. Well-being**	
	Well-being will be evaluated through measures such as:
	> Productivity through the assessment of income and employment
	> Quality of health life (SF-12 questionnaire).
	> Food insecurity (EBIA-5 questionnaire)
	> Loss of goods, services, and income due to flooding and landslides
**7. Long-term child development**	
	> Global and specific development in gross and fine motor coordination, communication, problem-solving and personal-social domains (ASQ-3 questionnaire)
	> Performance evaluation in Portuguese and Mathematics tests of Brazilian elementary education (Andres)
	> Anthropometric measures

**8. Enteric diseases**	
	The self-reported gastrointestinal infection will be evaluated through caregiver-reported symptom data in the last 7 days including:<br>> Diarrhoea (≥3 loose or liquid stools in a 24h period)
	> Vomiting
	> Abdominal pain
	> Refusal to eat
	> Hygiene practices
**9. Arboviral diseases**	
	> Accumulation of water in containers after rain
	> Presence of mosquitoes at home
	> Main time of the presence of mosquitoes at home
	> Diagnosis of an arbovirus in the last 6 months

Supplementary Figure 1. Schematic layout of the design of the conventional and simplified sewerage systems.

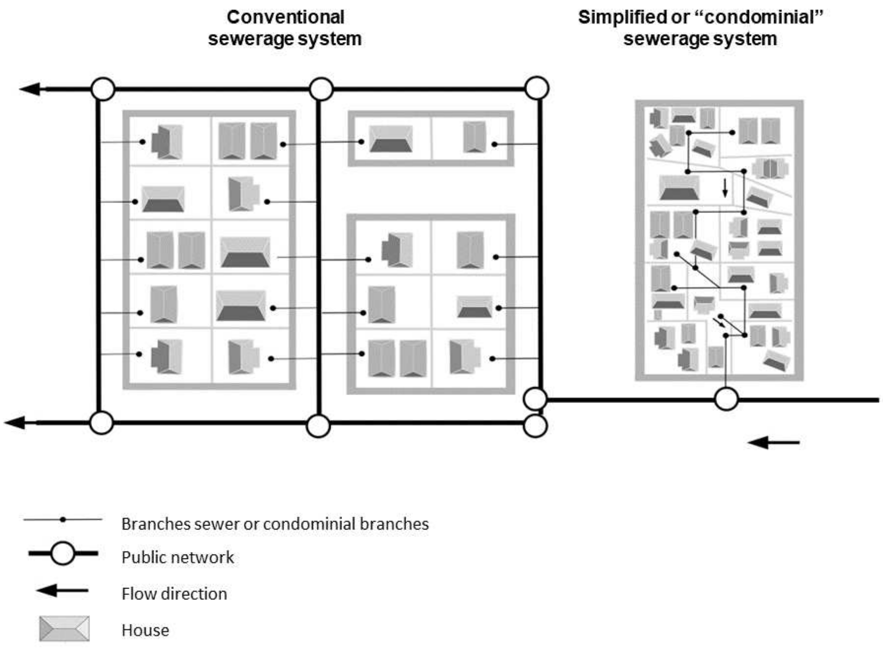

Supplement: Supplementary data [file bmjopen-2022-065009supp001.pdf]
