# Supplementary material for: Simplified sewerage to prevent urban leptospirosis transmission: a cluster non-randomised controlled trial protocol in disadvantaged urban communities of Salvador, Brazil
Source: BMJ Open. 2023 Jun 23;13(6):e065009. doi: 10.1136/bmjopen-2022-065009 (PMC10314607; doi:10.1136/bmjopen-2022-065009)
Supplement: Author's manuscript [file bmjopen-2022-065009.draft_revisions.pdf]

BMJ Open is committed to open peer review. As part of this commitment we make the peer review history of every article we publish publicly available.

When an article is published we post the peer reviewers' comments and the authors' responses online. We also post the versions of the paper that were used during peer review. These are the versions that the peer review comments apply to.

The versions of the paper that follow are the versions that were submitted during the peer review process. They are not the versions of record or the final published versions. They should not be cited or distributed as the published version of this manuscript.

BMJ Open is an open access journal and the full, final, typeset and author-corrected version of record of the manuscript is available on our site with no access controls, subscription charges or pay-per-view fees (http://bmjopen.bmj.com).

If you have any questions on BMJ Open's open peer review process please email info.bmjopen@bmj.com

BMJ Open

Simplified sewerage to prevent urban leptospirosis transmission: a cluster non-randomized controlled trial protocol in disadvantaged urban communities of Salvador, Brazil

Journal:	*BMJ Open*
Manuscript ID	bmjopen-2022-065009
Article Type:	Protocol
Date Submitted by the Author:	24-May-2022
Complete List of Authors:	Cremonese, Cleber; Federal University of Bahia,<br>SOUZA, FABIO; Universidade Federal da Bahia<br>Palma, Fabiana ; Federal University of Bahia<br>Sodré, Jonatas Fernandes; Federal University of Bahia<br>Ribeiro, Priscyla dos Santos ; Federal University of Bahia<br>Santana, Juliet ; Federal University of Bahia<br>Coelho, Rachel Helena; Federal University of Bahia<br>Ticona, Juan P. Aguilar ; Federal University of Bahia<br>Nazaré, Romero ; Fundação Oswaldo Cruz<br>de Oliveira, Daiana ; Federal University of Bahia<br>Silva, Cainã ; Federal University of Bahia<br>Eyre, Max ; Lancaster University<br>Knee, Jackie ; London School of Hygiene and Tropical Medicine Faculty of Infectious and Tropical Diseases<br>Ristow, Paula ; Federal University of Bahia<br>Stauber, Christine; Georgia State University<br>López, Yeimi Alexandra; Federal University of Bahia<br>Giorgi, Emanuele; Lancaster University Lancaster Medical School,<br>Diggle, Peter; Lancaster University, Faculty of Health and Medicine; University of Liverpool, Epidemiology and Population Health<br>Reis, Mitermayer Galvão ; Fundação Oswaldo Cruz<br>Cumming, Oliver; London School of Hygiene and Tropical Medicine, Department of Disease Control<br>Ko, Albert; Yale University,<br>Costa, Federico ; Federal University of Bahia
Keywords:	EPIDEMIOLOGY, Public health < INFECTIOUS DISEASES, BACTERIOLOGY

SCHOLARONE™
Manuscripts

Title: Simplified sewerage to prevent urban leptospirosis transmission: a cluster non-randomized controlled trial protocol in disadvantaged urban communities of Salvador, Brazil

Author affiliations

Cleber Cremonese[1], Fábio Neves Souza[1,2,3], Fabiana Almerinda Gonçalves Palma[1,2], Jonatas Fernandes Araújo Sodré[1], Priscyla dos Santos Ribeiro[4], Juliet Oliveira Santana[2], Rachel Helena Coelho[5], Juan P. Aguilar Ticona[1,2], Romero Nazaré[2], Daiana de Oliveira[1,2], Cainã Queiroz Silva[1], Max Eyre[6], Jackie Knee[7], Paula Ristow[3], Christine Stauber[8], Yeimi Alexandra Alzate López[1], Emanuele Giorgi[6], Peter John Diggle[6], , Mitermayer Galvão dos Reis[2,9], Oliver Cumming[7], Albert I. Ko[2,10] and Federico Costa[1,2,6,10]

[1]Institute of Collective Health, Federal University of Bahia, 40110-040, Salvador, Bahia, Brazil

[2]Instituto Gonçalo Moniz, Fundação Oswaldo Cruz, Ministério da Saúde, Rua Waldemar Falcão, 121, Salvador, Bahia, Brasil

[3]Institute of Biology, Federal University of Bahia, Ondina Campus, 40170-115, Salvador, Bahia, Brazil

[4]Institute of Biological Science, Federal University of Minas Gerais, Belo Horizonte, Brazil

[5]Faculty of Economics, Federal University of Bahia, Dois de Julho, Salvador, 40070-010, Salvador, Bahia, Brazil

[6]Lancaster Medical School, Lancaster University, Lancaster, LA1 4YW, UK

[7]London School of Hygiene & Tropical Medicine, Faculty of Infectious Tropical Diseases, Disease Control Department, United Kingdom

[8]Department of Population Health Sciences, School of Public Health, Georgia State University, Atlanta, GA, USA

[9]Faculty of Medicine of Bahia, Federal University of Bahia, Praça Conselheiro Almeida Couto, s/n - Largo do Terreiro de Jesus, Salvador, BA 40025-010, Salvador, Bahia, Brazil

For peer review only

[10]Department of Epidemiology of Microbial Diseases, School of Public Health, Yale University, 60 College Street, P.O. Box 208034, New Haven, CT, USA

Correspondence to: Cleber Cremonese (cleber.cremonese@gmail.com) and Federico Costa (federico.costa@ufba.br)

For peer review only

ABSTRACT

Introduction

Leptospirosis is a globally distributed zoonotic and environmentally mediated disease that has emerged as a major health problem in urban slums in developing countries. Its etiological agent is bacteria of the genus *Leptospira,* which are mainly spread in the urine of infected rodents, especially in an environment where adequate sanitation facilities are lacking and it known that open sewers are key transmission sources of the disease. Therefore, we aim to evaluate the effectiveness of a simplified sewerage intervention in reducing the risk of exposure to contaminated environments and *Leptospira* infection and to characterize the transmission mechanisms involved.

Methods and Analysis

This matched quasi-experimental study-design using non-randomized intervention and control clusters was designed to assess the effectiveness of an urban simplified sewerage intervention in the low-income communities of Salvador, Brazil. The intervention consists of household-level piped sewerage connections and community engagement and public involvement activities. A cohort of 1,400 adult participants will be recruited and grouped into eight clusters consisting of four matched intervention-control pairs with approximately 175 individuals in each cluster in baseline. The primary outcome is the seroincidence of *Leptospira* infection assessed through five serological measurements: one pre-intervention (baseline) and four post-intervention. As a secondary outcome, we will assess *Leptospira* load in soil, before and after the intervention. We will also assess *Leptospira* exposures before and after the intervention, through transmission modeling, accounting for residents' movement, contact with flooding, contaminated soil and water, and rat infestation, to examine whether and how routes of exposure for *Leptospira* change following the introduction of sanitation.

Ethics and dissemination

This study protocol has been reviewed and approved by the ethics boards at the Federal University of Bahia and the Brazilian National Research Ethics Committee. Results will be

For peer review only

disseminated through peer-reviewed publications and presentations to implementers, researchers, and participating communities.

Trial registration number: Brazilian Clinical Trials Registry, RCT trial RBR-8cjjpgm.

STRENGTHS AND LIMITATIONS OF THIS STUDY

- The first rigorous controlled intervention study for the effect of sanitation on *Leptospira* transmission. Includes several complementary interdisciplinary approaches, study designs and outcomes to progressively understand the mechanism for which simplified sewer coverage protects from pathogen exposure, *Leptospira* environmental contamination, and *Leptospira* infection.
- This study has the potential to guide future sanitation policies in communities with similar socio-economic status and environmental contexts.
- This is a cluster-based non-randomized controlled trial with intervention clusters receiving a simplified sanitation intervention and matched control clusters no intervention.
- We aim to recruit and follow 1,400 adults' participants across intervention and control clusters for 3 years. The serological surveys include measurement at baseline to assess the prevalence of *Leptospira* before the intervention, and 5 post-intervention serological surveys to obtain 4 seroincidence outcomes.
- The alliance between the natural and social sciences and the potential that it has to deepen community perceptions about leptospirosis and interventions before and after, as well as involvement in it in some decision-making processes in the improvement of their health, wellbeing, and sanitation.

For peer review only

INTRODUCTION

Globally, leptospirosis is a leading zoonotic disease in terms of morbidity and mortality [1], causing >1 million cases and >50,000 deaths yearly [1–3]. This zoonosis is an environmental and sanitation-related disease [4] characterized by annual epidemics during periods of increased rainfall and flooding [5,6]. Infection occurs frequently following exposure to water and soil that has been contaminated with the urine of infected animals [7,8]. Leptospirosis is an emerging disease in urban areas of most low- and middle-income countries (LMICs) [1], where a significant fraction of the population lives in disadvantaged urban communities (often called slums or *favelas* in Brazil) [9]. In those settings, poverty, precarious housing conditions, overpopulation, and lack of basic sanitation enable a high density of rats (the main urban reservoir hosts [10–13]) and high leptospirosis transmission [14,15]. As the population living in urban disadvantaged communities will double to 2 billion by 2025 [9] the burden of leptospirosis is expected to increase.

Public health intervention strategies to prevent leptospirosis transmission are essential and must target the infrastructure deficiencies to prevent or diminish human exposure to environmental contamination sources. Governmental interventions focused on improving sanitation are critical for reducing the burden of leptospirosis as well as other diseases, like diarrhea [4,16]. However, these interventions, especially in disadvantaged urban communities, are not a priority or foreseeable soon considering the high cost of providing and maintaining such sanitation infrastructure. Exclusion of the disadvantaged urban communities' health issues in the government's political agenda [17,18], contributes to historical and structural disparities. Brazil, since the eighties, has been recognized as a global leader in the development of cost-effective water and sanitation systems for densely populated urban settings [19,20]. These systems, called simplified or “condominial” water and sewerage systems, are designed to be installed in densely populated, informal communities that are often situated in the kind of hilly terrain, common in Brazilian favela communities. The simplified sewerage approach is unique because it redefines the service unit as a community block rather than an individual household. It uses smaller pipes and inspection chambers (or cleanouts) at key junctions to facilitate installation and maintenance [19,20]. The system design reduces costs by decreasing the length of the trenches needed for sewerage lines and by using smaller pipes made from less expensive materials such as PVC (polyvinyl chloride). The installation of this alternative system can cost 50-70% less than a

For peer review only

conventional system [19]. The lower costs associated with the installation of simplified systems makes these systems a more feasible alternative to conventional sewer systems in poor, resource-constrained settings. However, the simplified sewerage approach requires intensive community mobilization and engagement [21] as it is residents who take on leadership roles and have responsibility for decision-making.

Health impact evaluations of simplified sewerage approach in preventing disease has previously been limited to diarrheal diseases [22]. The evaluation of such interventions is challenging because of methodological problems such as cost and time of implementation, the requirement of large sample sizes, difficulties with randomization of the intervention allocation, and confounding factors (e.g. rodent infestation and Social-Economic Status). Contact with environments contaminated with sewer overflow or residents' exposure to open sewerage near the household can influence the degree of individual exposure to *Leptospira* bacteria[14]. Humans exposure to *Leptospira* contamination through poor sanitation is mediated by individual behavior, socioeconomic and environmental factors, rainfall, and flooding [4]. Therefore, the mechanisms of *Leptospira* transmission have different but important components of the environmental transmission route, for which their relative contribution is unknown because of confounding factors. Only studies that integrate a rigorous characterization of contamination sources (*Leptospira* load) and residents' movement could separate their effects and understand how source reduction interventions impact those transmission mechanisms. Determining the contribution of each of these mechanisms to *Leptospira* infection would help us to simultaneously understand the relationships and pathways that drive transmission [4]. This information is key to making interventions generalizable to other environmentally transmitted diseases that affect urban disadvantaged populations.

In this protocol, we describe an evaluation of a simplified sewerage intervention and community engagement with a quasi-experimental study-design using non-randomized intervention and control clusters. We aim to assess the longitudinal impact of a simplified sewerage intervention on the incidence and transmission mechanisms of *Leptospira* infection. In addition, we intend to investigate the impact of these interventions on reducing other water and environmental-related transmissible diseases.

Research aims and Hypotheses

For peer review only - http://bmjopen.bmj.com/site/about/guidelines.xhtml

We hypothesize that the combination of a simplified sewerage infrastructure and community engagement activities will reduce the risk of *Leptospira* infection among disadvantaged urban communities in Salvador, Brazil. We further hypothesize that this approach will be effective in reducing: 1) frequency of human contact with open sewerage contaminated with pathogenic *Leptospira*; 2) rat infestation; and 3) *Leptospira* load in the soil around the simplified sewerage**.** We aim to evaluate the efficacy of a sewerage intervention on reducing *Leptospira* infection and other health outcomes related to sanitation in disadvantaged urban communities in Brazil. Our specific objectives are to: (1) to evaluate prospectively the effectiveness of a simplified sewerage intervention in reducing the risk of *Leptospira* infection; (2) to determine the mechanisms by which the simplified sewerage reduce direct human contact with environmental pathogen load in urban slums. **Figure 1** provides further details on our hypotheses concerning the effects of the sanitation intervention on the transmission mechanisms of urban leptospirosis. We conceptualize this protocol as focusing on the sanitation intervention to decrease leptospirosis; however, this study protocol generates rich opportunities to evaluate other health outcomes. Therefore, we additionally aim to investigate the effects of the sewerage intervention on childhood exposure to a range of enteropathogens, and long-term child neurodevelopment and anthropometry. As exploratory outcomes, we will also investigate the effect of the sewerage intervention on the reduction of risk of arbovirus infectious diseases.

METHODS AND ANALYSIS

Overview of study design

This is an intervention trial with a cluster non-randomised controlled trial, with eight clusters (four intervention and four control), including longitudinal serological follow-up of 1,400 adults' residents from disadvantaged urban communities at high risk for leptospirosis transmission in Salvador, Bahia, Brazil. The primary outcome will be *Leptospira* infection. Each pair of intervention and control clusters will be allocated in distinct hydrological sub-basins with distances from 1,000 to 1,500 meters apart. Clusters will be matched on socio-economic status (SES), topographic or hydrologic characteristics, population density, and *Leptospira* seroprevalence. The intervention aims at a combination of sanitation (simplified sewerage intervention) and community engagement activities.

For peer review only

Study settings

Salvador is the third largest city in Brazil with a population of 2.9 million residents[23]. Using a surveillance database of active leptospirosis in the study setting between 1996 and 2018, we performed a population-based, adjusted Kernel analysis among geo-localized records of households with leptospirosis cases to identify high risk area corresponding to 8.06% of the total area of the city[15,24]. We selected eight clusters (four intervention and four control) located in these areas. The intervention clusters will be located in areas where the provision of simplified sewerage and community engagement is planned. Moreover, the choice of the control group depended on the implementer's information regarding non-intervention planned in the area in the short term (5 years-period). Geographic location of intervention and control clusters is detailed in **Figure 2**.

Description of the Sewerage intervention

During 2022 and 2023, the sewerage intervention (simplified sewerage intervention) will be implemented by the Public Water and Sanitation Bahia State Company (*Empresa Baiana de Águas e Saneamento*) in different urban locations in Salvador, Brazil. The expansion of the sanitation coverage in the city has generated an opportunity to assess the longitudinal effectiveness of the intervention on the transmission of sanitation-related diseases, such as leptospirosis. The company is government-owned, with responsibilities for managing, monitoring, and distributing water and sanitation services throughout the state of Bahia. The costs, management, and entire execution of the work will be the responsibility of the water and sanitation company, while the team of researchers will only monitor the development of the work.

This simplified sewerage intervention is characterized by receiving the sewage generated by a cluster of houses instead of individual houses. In conventional sewerage system designs, each household is connected directly to a principal line while in the sewer simplified system households are connected to branch lines within each block [22] **(Supplementary figure 1)**.

The sanitation company will carry out the sewerage network works, which include the installation of a 150 mm primary pipe at the bottom of the residential lot, bordering a watercourse. Households are connected to each other, and the connection to the main network. Thus, the intervention is

For peer review only

204 expected to increase sanitation coverage in the study clusters by expanding the collection network. These interventions would exclusively be of a sewerage nature, with no complementary functions such as local drainage. Importantly, the intervention also includes community participation in the action designs and implementation.

208 **Community engagement**

209 Simplified sewerage includes different degrees of social participation. We recognize that the intervention in sanitary sewerage impacts the local environment and the communities' perception is affected by the sanitation interventions. Problems and failures related to sewerage intervention have been reported as mainly due to the difficulties in the relationship between the technicians who accompany the works and the communities, one of which is that community participation is reduced only to the maintenance of the sewerage[21].

215 Faced with this complexity related to urban sanitation, the community engagement processes that will be implemented in this study are focused on approaches that would stimulate community mobilization to participate in the research processes, popular health education actions, and in the evaluation of the implementation of sewerage intervention. Popular Health Education (PHE) actions will be oriented toward disease prevention and health promotion of the population. The PHE is a "set of practices targeted at individuals and the community"[25,26], and in this study intended to contribute to community empowerment and support for the management of sewerage intervention. The community engagement activities would be implemented in stages and with different stakeholders, including the community leaders, residents, and a multidisciplinary team of researchers including epidemiologists, ecologists, sanitation engineers, anthropologists, environmental microbiologists, geographers, economists, statisticians, etc. The study, therefore, aims to strengthen community participation in the decision-making in the management of sewerage intervention, and also support the involvement of residents in activities research such as (1) Community and institutional mobilization; (2) Collaborative mapping; and (3) Popular Health Education Actions. Moreover, we will perform community ethnographic to support the expansion of the understanding of the social and cultural impacts caused by the intervention before and after its realization, the environmental impacts, and the mobilization processes.

232 **Study population and sample size**

For peer review only

To evaluate the effectiveness of the simplified sewerage intervention in reducing the risk of *Leptospira* infection, we plan to recruit an estimated 1,400 participants aged ≥18 years, residing in the selected clusters (approximately 175 adults per cluster in baseline). To evaluate the intervention effect on secondary (sewerage contact, environmental pathogen load, long-term child development) and exploratory (arbovirus infections, diarrheal disease, and enteric infection) outcomes, we plan to increase our sample size by 420 (30%) participants, aged between 6 months and 17 years, totaling 1,840 people. All participants from both intervention and control clusters will be followed over a period of three years after the sewerage intervention and a baseline prior to the intervention **(Figure 2)**. In the flow diagram, we provide a timeline and trial study phases in detail (**Figure 3)**.

The sample size was estimated using data on *Leptospira* infection from a previous leptospirosis cohort study in Salvador, Brazil [14]. We divided the previous leptospirosis cohort study [14] into areas of similar size to that the clusters used trial and calculated the between-cluster variation. Of 1,840 individuals participating in the previous cohort, 142 (7.9%) experienced leptospiral infection (k/ICC=0.03). Power calculations are based on a cluster-level using effect sizes (% decrease in incidence between intervention and control clusters) 40, 50, and 60%, assuming an infection rate of 0.08 per person-year. All sample size calculations used methods for matched cluster-randomized trials with the study structure of four matched pairs of clusters. Sample size estimates assumed a 12% annual drop-out rate across the three-year study period.

Control selection

Control clusters (CONT) will be selected in locations without with an incomplete sewerage sanitation system. Matching criteria for control clusters include: location in a geographic area that has similar environmental (elevation, hydrologic data) and social (income, education, population density) characteristics to the intervention clusters; and at a distance of 1,000 and 1,500 meters far from the intervention cluster. Areas that received major infrastructure interventions and

sanitation improvements in the last five years will be excluded from the selection frame for control clusters.

Eligibility and enrolment

Before the recruitment of study participants, a census will be carried out within the selected eight clusters to enumerate all residents from households within <40m distance from an open sewer. This would ensure that households and residents living in areas with a higher risk of leptospirosis transmission are included. At each census, the individual data (name, sex, age) and the geo-coordinates a primary household respondent would be collected. As a further step, we will randomly select population-based cohorts with 95 households (175 participants) per cluster. Residents will be eligible for inclusion in the study, if (1) they are 18 years of age or above, (2) have lived in the area for at least six months, (3) sleep in the household for at least 3 nights per week, and (4) consent to the study procedures and complete an informed consent form. However, residents will be excluded if: (1) they do not meet all of the inclusion criteria; (2) they cannot provide written informed consent to participate in the study, (3) they refuse or are unable to provide demographic information and biological sample; (4) they are with limited mental health problems or other problems that have implications for survey responses or participation in any other phase of research activity.

For secondary outcomes we will include all children and young between 6 months and 17 years of age will be included in the study **(Supplementary table 1)**. We will apply similar inclusion and exclusion criteria as described but excluding children for which their legal guardians do not provide written informed consent for their participation.

Outcomes

Primary outcome measure

The study trial will consist of three specifics phases: (1) baseline; (2) sanitation intervention implementation; (3) follow-up of the residents and evaluation of the sanitation intervention **(Figure 2)**. During the phase (1) a baseline survey will be conducted to characterize the demographics, socioeconomic status and *Leptospira* seroprevalence in each cluster. During the phase (3)

For peer review only

biannual serosurveys in the cohorts will be conducted to measure the primary outcome, *Leptospira* asymptomatic infection, and estimate the risk of infection during the dry and rainy seasons. The field team will collect blood samples from eligible participants with age ≥18 years. A blood sample (10mL) will be collected from each study participant and examined for *Leptospira* antigen using the microscopic agglutination test diagnostic (MAT) [4]. MAT is the standard test to determine *Leptospira* infection in longitudinal studies and *Leptospira* infection will be defined as a four-fold rise in MAT titer or seroconversion (negative to ≥1:50) between samples from consecutive serosurveys. We will use a panel of six reference strains: representing *L. interrogans* serovars Autumnalis strain Akiyami A, Canicola strain H. Utrecht IV and Icterohaemorrhagiae strain RGA, *L. borgpetersenii* serovar Ballum strain MUS 127, *L. kirschneri* serovar Cynopteri strain 3522C and Grippothyphosa strain Duysterand the two clinical isolates: *L. interrogans* serovar Copenhageni strain Fiocruz L1-130 (isolated locally in 1996) and *L. santarosai* serogroup Shermani strain LV3954 (isolated locally). This procedure had the same performance in identifying MAT seroconversion in our prospective studies [4,15] compared with the WHO recommended panel of 19 reference serovars. The screening will be performed with serum dilutions of 1:50-1:100. When agglutination is observed at 1:100, the sample will be titrated to determine the highest titer. As part of quality control procedures, two independent evaluations will be conducted for 8% of the samples and all samples from infected subjects.

Secondary outcomes

Environmental exposure secondary outcomes

Secondary outcomes for environmental effects will include microbiological assays to assess soil contamination with pathogenic *Leptospira*. The presence and concentration of pathogenic *Leptospira* will be evaluated in the eight clusters. A total of 68 soil samples and 15 water sewage samples will be collected in each cluster and transported refrigerated to the laboratory within six hours for processing. Sampling will take place in duplicate, with intervals of seven to nine days

between them. After processing, soil and sewage samples will be stored at -80°C until molecular assay. The presence and concentration of pathogenic *Leptospira* will be evaluated using a qPCR assay, targeting the *lipL32* gene. Samples will be considered positive when the two qPCR replicates show amplification before a Cq of 40 [8]. Environmental variables for rainfall, temperature, landscape (remote sensing using drones), flooding, and hydrological features will also be collected during rainy seasons before and at least three months immediately after the sewerage intervention.

Rats Survey

Rat abundance will be examined at each cluster using tracking boards to quantify the level of rat activity, before and after the interventions. The use of tracking boards to quantify rat presence and activity (as rat abundance proxy) will be performed, following previously described, validated, and optimized methods [27]. Tracking boards will be distributed in each cluster for two days in a row and will be scored daily. The overall level of rat presence for each cluster will be estimated based on the percentage of positive tracking boards and the intensity of marks on each board before and at least three months immediately after the sewerage intervention.

Human movement

Environmental exposure to *Leptospira* (i.e., proximity to open sewer and unpaved roads; housing conditions; and flooding) will be quantified by studying of the individual movement of the participants in each study cluster. Movement evaluations will be performed biannually, before and after the sewerage intervention. We will focus on a sub-cohort of 320 residents living in each of the eight study clusters, divided into four groups: young males (18–34 years), young females (18–34 years), old males (aged ≥35 years), and old females (aged ≥35 years). We will use GPS data loggers that can measure small-scale movement patterns [28,29].

Forty residents in each of the study clusters will be invited to use a GPS data logger IgotU® GT-600 device (Mobile Action Technology, New Taipei City, Taiwan) [30]. Additionally, we will obtain

For peer review only

335 information about movement and behavior through resident diaries and retrospective mapping
336 before and after the intervention. Diaries will provide qualitative information on the number of
337 hours inside/outside the study cluster and the household; contact duration and nature of the
338 contact (i.e., contact duration with and without use of boots) with contaminated sources; and the
339 activity performed (i.e., working, leisure, etc.) for 7 days. Retrospective mapping will provide
340 information on the past 24 hours' pathways traveled by the residents, and contacts with
341 contamination sources from the residents' perspective [29].

342 **Other outcomes**

343 *Human health and wellbeing*

344 The data will be collected pre and post-intervention to evaluate the impact on well-being between
345 arms. Other outcomes related to individual and household health and well-being include
346 subjective measures - self-reported general, physical and emotional well-being using the Short-
347 Form Health Survey-12 (SF-12) [31]; perceived living conditions questions; the short version (five
348 items) of the Brazilian Food Insecurity Scale [32] which measures the perception and experience
349 of hunger over the past 3 months; and objectives measures - productivity through employment
350 and income. We will also evaluate whether there will be a decrease in material and economic loss
351 measures due to floods and landslides -which often occur in the areas without adequate
352 sanitation.

353 *Long-term child development*

354 Child neurodevelopment will be evaluated using the Ages & Stages Questionnaires 3rd edition
355 (ASQ-3) for children aged 6 to 66 months and with the Brazilian National Assessment of School
356 Performance (ANRESC - *Avaliação Nacional do Rendimento Escolar)* for children older than 66
357 months, in the pre- and post-intervention period. ASQ-3 is a tool used to monitor the child's
358 developmental progress, it includes fine and gross motor, communication, problem-solving, and
359 personal-social domains [33]. ANRESC is part of the Brazilian Basic Education Assessment
360 System, which included the assessment of Math and Language skills [34]. The child development
361 assessment will also include measurements of weight, height, BMI and head circumference. We

For peer review only

will use the WHO Child Growth Standards and the results will be summarized using the Z-score [35].

Enteric diseases

To evaluate the impact of the intervention on enteric pathogen exposure among young children, we will collect stool and/or rectal swab samples from children aged 6 months to 5 years old in the pre-post-intervention periods. We will use a custom-designed TaqMan Array Card (TAC) to analyse samples for the presence of over 30 enteric pathogens, including viruses, bacteria, protozoa, and helminths. The TAC uses microfluidic technology to simultaneously perform 48 individual quantitative polymerase chain reactions per sample and has been previously validated and used for detection enteric pathogens in stool[36–38].

Arboviral diseases

We will evaluate the effectiveness of the sewerage intervention and the changes in landscape characteristics associated with them, in the spatiotemporal distribution of arbovirus (Dengue, Zika, Chikungunya) vector mosquitoes. We will carry out entomological investigations in private (participants' homes) and public areas with the collection of adult and immature mosquitoes by aspiration [39], with the verification of identified mosquitoes breeding sites, to measure their density. We will verify whether there is a spatiotemporal pattern in the presence and density of vector mosquitoes in the sampled regions, to allow us to investigate the possible factors that may be related to changes in the distribution of exposures before, during, and after the intervention process and whether these interventions have modified ecological indices for vector abundance.

Data Collection and management

Trained interviewers will administer specific questionnaires to participants through face-to-face interviews to collect general demographic information, socioeconomic data, exposure factors and individual or collective sanitation practices, and knowledge about leptospirosis and other diseases. In the case of children at home, a new questionnaire will be answered by the caregiver. Information will be collected on the child's general health status, development, and medical history and diseases **(Supplementary table 2)**.

For peer review only

389 All questioners and forms described in this protocol will be included in the Research Electronic Data Capture (REDCap) database. The REDCap web version as well as its mobile application will be used to record all data using mobile devices. A data management staff will keep track of the information in real time.

393 Patient and public involvement

394 All study subjects will be involved in the trial, answering questionnaires, providing biological samples, and involved in engagement activities as previously described in the section on community engagement. The sanitation intervention will be conducted by a governmental agency, and the implementation of this intervention involves dialogue with the residents of the communities. However, the study design and conduct of the trial of this intervention study will be taking place independently of public involvement. After each follow-up completion, research findings will be shared and disseminated in a group's meetings or visit households with key stakeholders.

402 Statistical analysis

403 Descriptive statistics will be employed to report the demographic, social, economic, or environmental characteristics between the study clusters (intervention and control clusters). For the primary outcome, we will apply quasi-Poisson regression to estimate the incidence rate ratio (IRR) of *Leptospira* infection. We will also evaluate the effect of the intervention on *Leptospira* infection risk using mixed-effects logistic regression models to account for clustering of the observations within study units and across repeated measures for individuals. We will model the overall effect of the intervention on infection risk while controlling for baseline sewerage and flooding exposure and potential confounding variables, such as age, sex, indicators of the socioeconomic level, and rainfall intensity during an annual period, which are presumably unrelated to the intervention **(Figure 1)**.

413 For the secondary outcome, we will conduct environmental analyses of *Leptospira* load between intervention and control clusters to compare environmental risk. To do this, we will model two leptospiral load outcomes, prevalence (binomial) and concentration (log-transformed), using generalized linear models with random effects to account for clustering within study units. The association between environmental contamination and hypothesized predictors of risk (rat

abundance, human movement, and ecological data) will also be explored in the model **(Figure 1)**.

Limitations and bias

The allocation is non-randomised with selection based on intervention criteria determined by the implementer so the risk of residual confounding cannot be excluded. The choice of the control group is also dependent on the implementer's information about the non-intervention in the area in the short term (5 years-period). To limit the potential for bias arising from our non-randomised design, we will select control group with as similar as possible socioeconomic and environmental characteristics to those of their matched intervention clusters.

We will perform the impact calculation through the double differentiation of the averages of the results found between the intervention and control clusters before and after the sewerage intervention. The mitigation of bias will be carried out through the control of fixed effects of time and location between different intervention and control clusters over the years of evaluation.

ETHICS AND DISSEMINATION

This project was evaluated and approved by a local ethics committee (CEP/ISC/UFBA) under number CAEE 32361820.7.0000.5030, and a national research ethics committee (CONEP) linked to the Brazilian Ministry of the Health under approval number 4.235.251. The trial study protocol was registered with the Brazilian Clinical Trials Registry, RCT trial RBR-8cjjpgm (https://ensaiosclinicos.gov.br/rg/RBR-8cjjpgm). All participants involved in the study will provide informed consent before any study activity, and the community leaders, previously identified by the team, will provide verbal consent before enrollment of the community in the trial. Participants aged 12 and 17 will sign an assent form, in addition to a participation consent form completed through their parents or legal guardians. Children under the age of 12 will have their consent signed by their parents or legal guardians only. The results of the trial will be presented at local and international meetings and submitted to peer-reviewed journals for publication. Results will also be shared directly with the participating communities. All individual information participants will be kept and published anonymously.

Authors' contributions

For peer review only - http://bmjopen.bmj.com/site/about/guidelines.xhtml

FC, CC, MGR, AK, YAL, PD, CS and OC contributed with project's main conceptual ideas; FC, ME, EG, and CC contributed to study design and statistical analysis plan; FNS, DO, FAP, CS, JFA, PSR, JOS, RHC, DS, RN, JPAT, JK, PR, FC, and CC developed field and laboratory protocols and implementation; FNS, FAP, CC, RHC, and FC developed data collection tools; FNS, FAP, JFA, RHC, PR, CC, and FC contributed to drafting the main manuscript. All authors contributed to editing and revising the manuscript.

Funding statement

This work was supported by Wellcome Trust grant number 102330/Z/13/Z. All project costs are jointly funded by Wellcome Trust and the Department of Health and Social Assistance (DHSC), through the National Institute for Health Research (NIHR), using the UK Official Development Assistance Fund (ODA).

Competing interest statement

Not applicable

Data sharing statement

The data collected with personal identifying data redacted will be available encoded for use by principal investigators on request following the publication of trial results. The funders have no role in the study design, data collection, and analysis, decision to publish, or preparation of manuscripts.

Acknowledgments

The authors gratefully acknowledge critical input on the study protocol and all the study participants. We acknowledge the partner institutions for their collaboration and dedication. We thank Patricia Lustosa, Ricardo Lustosa, and Mayara Carvalho for their help with the review and discussion of this protocol, Ana Maria Silva and George G. Machado for help with supplementary figure, and Hammed O. Mogaji for his help with manuscript proofing.

REFERENCES

1 Costa F, Hagan JE, Calcagno J, *et al.* Global Morbidity and Mortality of Leptospirosis: A Systematic Review. *PLoS Negl Trop Dis* 2015;**9**:e0003898.

For peer review only

doi:10.1371/journal.pntd.0003898

2 Ko AI, Goarant C, Picardeau M. Leptospira: the dawn of the molecular genetics era for an emerging zoonotic pathogen. *Nat Rev Microbiol* 2009;**7**:736–47. doi:10.1038/nrmicro2208

3 Bharti AR, Nally JE, Ricaldi JN, *et al.* Leptospirosis: a zoonotic disease of global importance. *Lancet Infect Dis* 2003;**3**:757–71. doi:10.1016/S1473-3099(03)00830-2

4 Khalil H, Santana R, de Oliveira D, *et al.* Poverty, sanitation, and Leptospira transmission pathways in residents from four Brazilian slums. *PLoS Negl Trop Dis* 2021;**15**:e0009256. doi:10.1371/journal.pntd.0009256

5 Ko AI, M GR, Cm RD, *et al.* Urban epidemic of severe leptospirosis in Brazil. Salvador Leptospirosis Study Group. *Lancet* 1999;**354**. doi:10.1016/s0140-6736(99)80012-9

6 Sarkar U, Nascimento SF, Barbosa R, *et al.* Population-based case-control investigation of risk factors for leptospirosis during an urban epidemic. *Am J Trop Med Hyg* 2002;**66**. doi:10.4269/ajtmh.2002.66.605

7 Bharti AR, Nally JE, Ricaldi JN, *et al.* Leptospirosis: a zoonotic disease of global importance. *Lancet Infect Dis* 2003;**3**:757–71. doi:10.1016/s1473-3099(03)00830-2

8 Casanovas-Massana A, Pedra GG, Wunder EA Jr, *et al.* Quantification of Leptospira interrogans Survival in Soil and Water Microcosms. *Appl Environ Microbiol* 2018;**84**. doi:10.1128/AEM.00507-18

9 United Nations Human Settlements Programme. *The Challenge of Slums: Global Report on Human Settlements, 2003*. UN-HABITAT 2003. https://books.google.com/books/about/The_Challenge_of_Slums.html?hl=&id=q4B4YvnUS7cC

10 Minter A, Diggle PJ, Costa F, *et al.* Evidence of multiple intraspecific transmission routes for Leptospira acquisition in Norway rats (Rattus norvegicus). *Epidemiol Infect* 2017;**145**:3438–48. doi:10.1017/S0950268817002539

11 Panti-May JA, Carvalho-Pereira TSA, Serrano S, *et al.* A Two-Year Ecological Study of Norway Rats (Rattus norvegicus) in a Brazilian Urban Slum. *PLoS One* 2016;**11**:e0152511. doi:10.1371/journal.pone.0152511

12 Costa F, Wunder EA Jr, De Oliveira D, *et al.* Patterns in Leptospira Shedding in Norway Rats (Rattus norvegicus) from Brazilian Slum Communities at High Risk of Disease Transmission. *PLoS Negl Trop Dis* 2015;**9**:e0003819. doi:10.1371/journal.pntd.0003819

13 Costa F, Porter FH, Rodrigues G, *et al.* Infections by Leptospira interrogans, Seoul virus, and Bartonella spp. among Norway rats (Rattus norvegicus) from the urban slum environment in Brazil. *Vector Borne Zoonotic Dis* 2014;**14**:33–40. doi:10.1089/vbz.2013.1378

14 Hagan JE, Moraga P, Costa F, *et al.* Spatiotemporal Determinants of Urban Leptospirosis Transmission: Four-Year Prospective Cohort Study of Slum Residents in Brazil. *PLoS Negl Trop Dis* 2016;**10**:e0004275. doi:10.1371/journal.pntd.0004275

15 Reis RB, Ribeiro GS, Felzemburgh RDM, *et al.* Impact of environment and social gradient on Leptospira infection in urban slums. *PLoS Negl Trop Dis* 2008;**2**:e228. doi:10.1371/journal.pntd.0000228

16 Barreto ML, Genser B, Strina A, *et al.* Effect of city-wide sanitation programme on reduction in rate of childhood diarrhoea in northeast Brazil: assessment by two cohort studies. *Lancet* 2007;**370**:1622–8. doi:10.1016/S0140-6736(07)61638-9

17 Bolay J-C. Slums and Urban Development: Questions on Society and Globalisation. *The European Journal of Development Research* 2006;**18**:284–98.

For peer review only

doi:10.1080/09578810600709492

18 Staff UNHSP, Programme UNHS. *The challenge of slums: global report on human settlements, 2003*. UN-HABITAT 2003.

19 Melo JC. The experience of condominial water and sewerage systems in Brazil: case studies from Brasilia, Salvador and Parauebas. The World Bank 2005.

20 Melo JC. Sistema Condominial: uma resposta ao desafio da universalização do saneamento. *Ministério das Cidades* 2008.

21 Nance E, Ortolano L. Community Participation in Urban Sanitation: Experiences in Northeastern Brazil. *Journal of Planning Education and Research* 2007;**26**:284–300. doi:10.1177/0739456X06295028

22 Hubbard B, Sarisky J, Gelting R, *et al.* A community demand-driven approach toward sustainable water and sanitation infrastructure development. *Int J Hyg Environ Health* 2011;**214**:326–34. doi:10.1016/j.ijheh.2011.05.005

23 IBGE. Estimated Population 2017. Cities, Bahía, Salvador. https://cidades.ibge.gov.br/brasil/ba/salvador/panorama (accessed 11 Apr 2022).

24 Srihera R, Stute W. Kernel adjusted density estimation. *Stat Probab Lett* 2011;**81**:571–9. doi:10.1016/j.spl.2011.01.013

25 Seabra CAM, Xavier SPL, Sampaio YPC, *et al.* Health education as a strategy for the promotion of the health of the elderly: an integrative review. Revista Brasileira de Geriatria e Gerontologia. 2019;**22**. doi:10.1590/1981-22562019022.190022

26 Falkenberg MB, Mendes T de PL, Moraes EP de, *et al.* Educação em saúde e educação na saúde: conceitos e implicações para a saúde coletiva. *Ciênc saúde coletiva* 2014;**19**:847–52. doi:10.1590/1413-81232014193.01572013

27 Hacker KP, Minter A, Begon M, *et al.* A Comparative Assessment of Track Plates to Quantify Fine Scale Variations in the Relative Abundance of Norway Rats in Urban Slums. *Urban Ecosyst* 2016;**19**:561–75. doi:10.1007/s11252-015-0519-8

28 Vazquez-Prokopec GM, Stoddard ST, Paz-Soldan V, *et al.* Usefulness of commercially available GPS data-loggers for tracking human movement and exposure to dengue virus. International Journal of Health Geographics. 2009;**8**:68. doi:10.1186/1476-072x-8-68

29 Owers KA, Odetunde J, de Matos RB, *et al.* Fine-scale GPS tracking to quantify human movement patterns and exposure to leptospires in the urban slum environment. *PLoS Negl Trop Dis* 2018;**12**:e0006752. doi:10.1371/journal.pntd.0006752

30 Morris G, Conner LM. Assessment of accuracy, fix success rate, and use of estimated horizontal position error (EHPE) to filter inaccurate data collected by a common commercially available GPS logger. *PLoS One* 2017;**12**:e0189020. doi:10.1371/journal.pone.0189020

31 Ware J Jr, Kosinski M, Keller SD. A 12-Item Short-Form Health Survey: construction of scales and preliminary tests of reliability and validity. *Med Care* 1996;**34**:220–33. doi:10.1097/00005650-199603000-00003

32 Santos LPD, Lindemann IL, Motta JVDS, *et al.* Proposal of a short-form version of the Brazilian food insecurity scale. *Rev Saude Publica* 2014;**48**:783–9. doi:10.1590/s0034-8910.2014048005195

33 Filgueiras A, Pires P, Maissonette S, *et al.* Psychometric properties of the Brazilian-adapted version of the Ages and Stages Questionnaire in public child daycare centers. *Early Hum Dev* 2013;**89**:561–76. doi:10.1016/j.earlhumdev.2013.02.005

34 Brasil M, da Aneb IM. da Anresc 2015. *Brasília: Inep* 2017.

For peer review only

35 de Onis M, Garza C, Victora CG, *et al.* The WHO Multicentre Growth Reference Study: planning, study design, and methodology. *Food Nutr Bull* 2004;**25**:S15–26. doi:10.1177/15648265040251S103

36 Platts-Mills JA, Liu J, Rogawski ET, *et al.* Use of quantitative molecular diagnostic methods to assess the aetiology, burden, and clinical characteristics of diarrhoea in children in low-resource settings: a reanalysis of the MAL-ED cohort study. *The Lancet Global Health* 2018;**6**:e1309–18.https://www.sciencedirect.com/science/article/pii/S2214109X18303498

37 Liu J, Platts-Mills JA, Juma J, *et al.* Use of quantitative molecular diagnostic methods to identify causes of diarrhoea in children: a reanalysis of the GEMS case-control study. *Lancet* 2016;**388**:1291–301. doi:10.1016/S0140-6736(16)31529-X

38 Liu J, Gratz J, Amour C, *et al.* Optimization of Quantitative PCR Methods for Enteropathogen Detection. *PLoS One* 2016;**11**:e0158199. doi:10.1371/journal.pone.0158199

39 Vazquez-Prokopec GM, Galvin WA. A new, cost-effective, battery-powered aspirator for adult mosquito collections. *Journal of medical* Published Online First: 2009.https://academic.oup.com/jme/article-abstract/46/6/1256/921254

For peer review only

FIGURES

Figure 1- Rationale, hypothesis, and outcomes assessed in the study. We hypothesize that sanitation intervention will improve the environmental conditions and change the behavior of humans, resulting in both direct and indirect impacts on the incidence of leptospirosis and other diseases human and well-being.

Figure 2- Study clusters in the city of Salvador, Brazil. Altitude and sampling clusters (**A**). Example of baseline sanitation conditions of the study cluster (**B**). In (**C**) Consort flow diagram by intervention and control cluster, data collection times and type of activities.

Figure 3- Overview of the study procedures, the timeline for study, and data collection. **Note 1**: Each cluster of the study will be formed by a cluster with intervention vs. Control. The period of activities for each serological survey will take place at the time described in the figure (Allocation, Recruitment, and Baseline). **Note 2**: Update years 1 to 5 will be realized after receipt of the water and sanitation company schedule. SES: Socio-economic status.

For peer review only

For peer review only

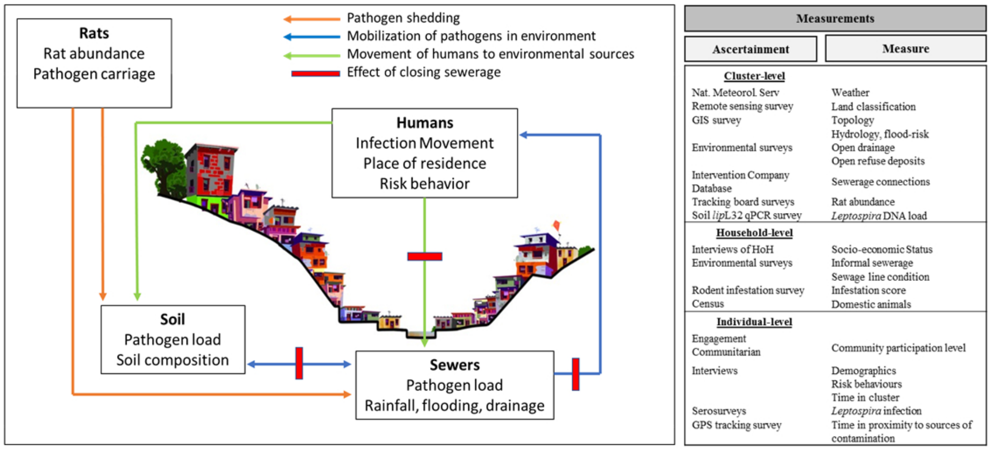

Figure 1- Rationale, hypothesis, and outcomes assessed in the study. We hypothesize that sanitation intervention will improve the environmental conditions and change the behavior of humans, resulting in both direct and indirect impacts on the incidence of leptospirosis and other diseases human and well-being.

242x108mm (120 x 120 DPI)

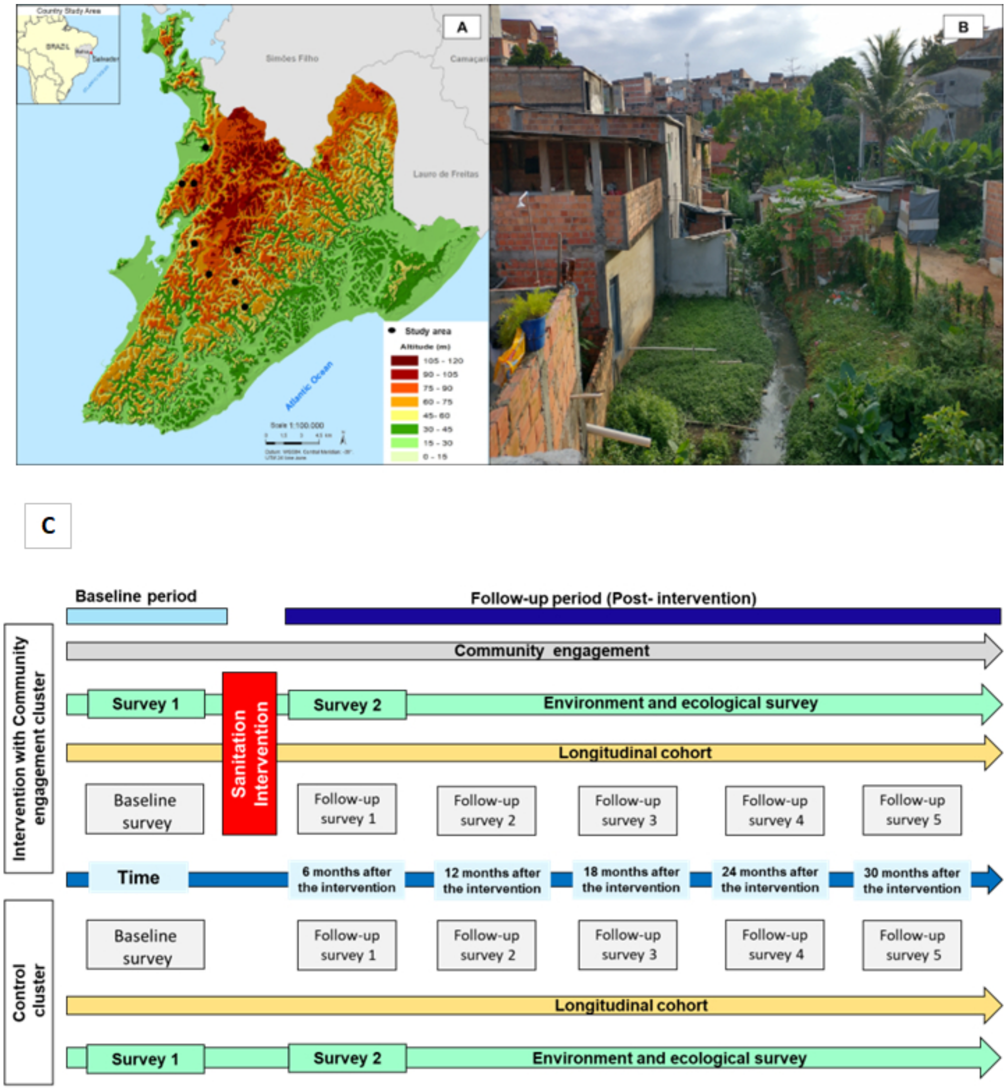

Figure 2- Study clusters in the city of Salvador, Brazil. Altitude and sampling clusters (A). Example of baseline sanitation conditions of the study cluster (B). In (C) Consort flow diagram by intervention and control cluster, data collection times and type of activities.

151x165mm (120 x 120 DPI)

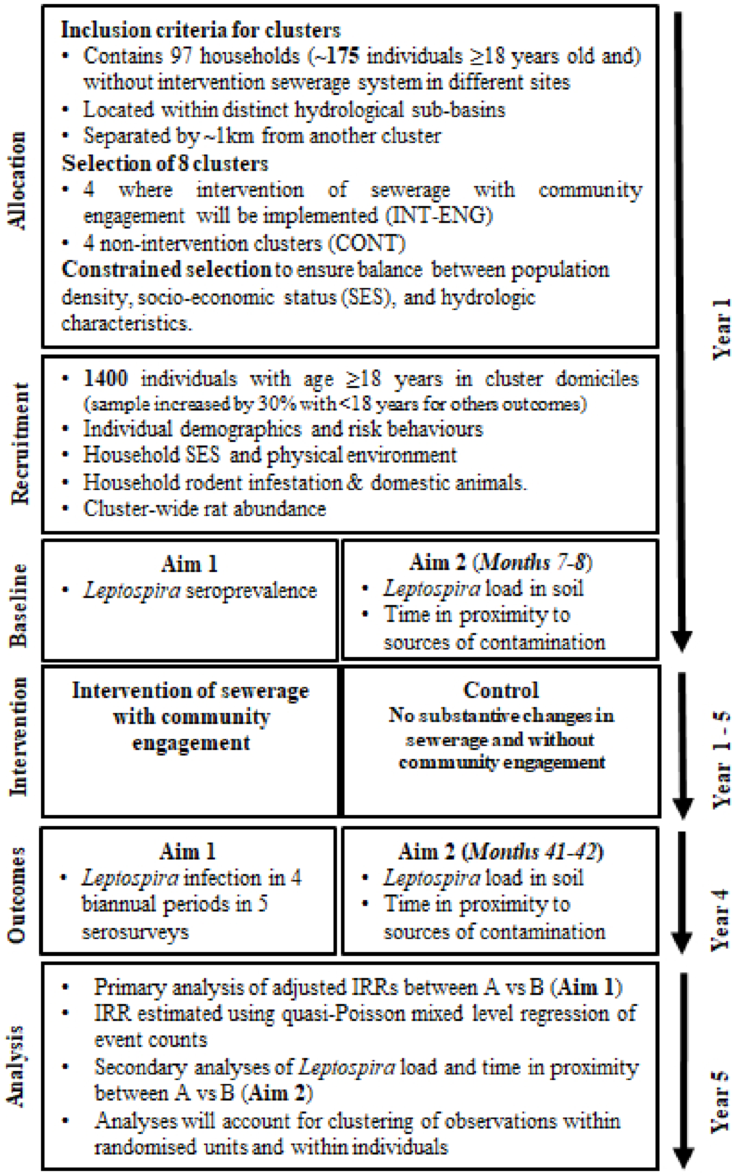

Figure 3- Overview of the study procedures, the timeline for study, and data collection. Note 1: Each cluster of the study will be formed by a cluster with intervention vs. Control. The period of activities for each serological survey will take place at the time described in the figure (Allocation, Recruitment, and Baseline). Note 2: Update years 1 to 5 will be realized after receipt of the water and sanitation company schedule. SES: Socio-economic status.

96x136mm (120 x 120 DPI)

Supplementary material

Supplementary Table 1. Specific age range for each outcome.

Outcome	Age group
Leptospirosis, preventive practice, well-being, perceptions of residents	≥ 18 years
Arboviral diseases	≥ 6 months
Long-term infant development	≥ 6 to ≤ 66 months
Enteric diseases	≥ 6 to ≤ 66 months

For peer review only

Supplementary Table 2. Primary and secondary outcomes baseline in assessed in this study protocol, Salvador, Brazil, 2022

Outcomes of interest	Operational definition
**1. Individual *Leptospira* infection**	
	> *Leptospira* infection defined as a four-fold rise in microscopic agglutination test (MAT) titer or seroconversion (negative to ≥1:50) between samples from consecutive serosurveys.
**2. Individual risk for *Leptospira* exposure**	
	> Walk-in flooding in the neighborhood<br>> Walk barefoot outside the household<br>> Need to unclog sewerage<br>> Perception of vulnerability to leptospirosis<br>> Perception of the severity of the leptospirosis disease<br>> Perception of the presence of rats near the house
**3. Environmental for *Leptospira* exposure**	
	> Proximity and contact frequency to open sewer, trash, and other exposures to environmental sources<br>> Sewage infrastructure at home and in the community<br>> Flooding in the community and inside the home<br>> Floor and road conditions<br>> Accumulation of garbage near the house<br>> Presence and concentration of *Leptospira* load in the soil
**4. Sanitation-related knowledge and practice**	
	Consequences of lack or poor sanitation as:<br>> Structural problems<br>> Major related diseases<br>> Institutions responsible for providing sanitation in the community<br>> Practice related to sanitation in-home and community
**5. Social Capital**	
	> Self-Assessment of coexistence and trust with neighbors.<br>> Perception of neighbors' willingness to help each other<br>> Social, leisure, and community support activities practiced<br>> Willingness to continue living in the community
**6. Well-being**	
	Well-being will be evaluated through measures such as:<br>> Productivity through the assessment of income and employment<br>> Quality of health life (SF-12 questionnaire).<br>> Food insecurity (EBIA-5 questionnaire)<br>> Loss of goods, services, and income due to flooding and landslides
**7. Long-term child development**	
	> Global and specific development in gross and fine motor coordination, communication, problem-solving and personal-social domains (ASQ-3 questionnaire)<br>> Performance evaluation in Portuguese and Mathematics tests of Brazilian elementary education (Andres)<br>> Anthropometric measures

For peer review only

**8. Enteric diseases**	
	The self-reported gastrointestinal infection will be evaluated through caregiver-reported symptom data in the last 7 days including:<br>> Diarrhoea (≥3 loose or liquid stools in a 24h period)<br>> Vomiting<br>> Abdominal pain<br>> Refusal to eat<br>> Hygiene practices
**9. Arboviral diseases**	
	> Accumulation of water in containers after rain<br>> Presence of mosquitoes at home<br>> Main time of the presence of mosquitoes at home<br>> Diagnosis of an arbovirus in the last 6 months

For peer review only

Supplementary Figure 1. Schematic layout of the design of the conventional and simplified sewerage systems.

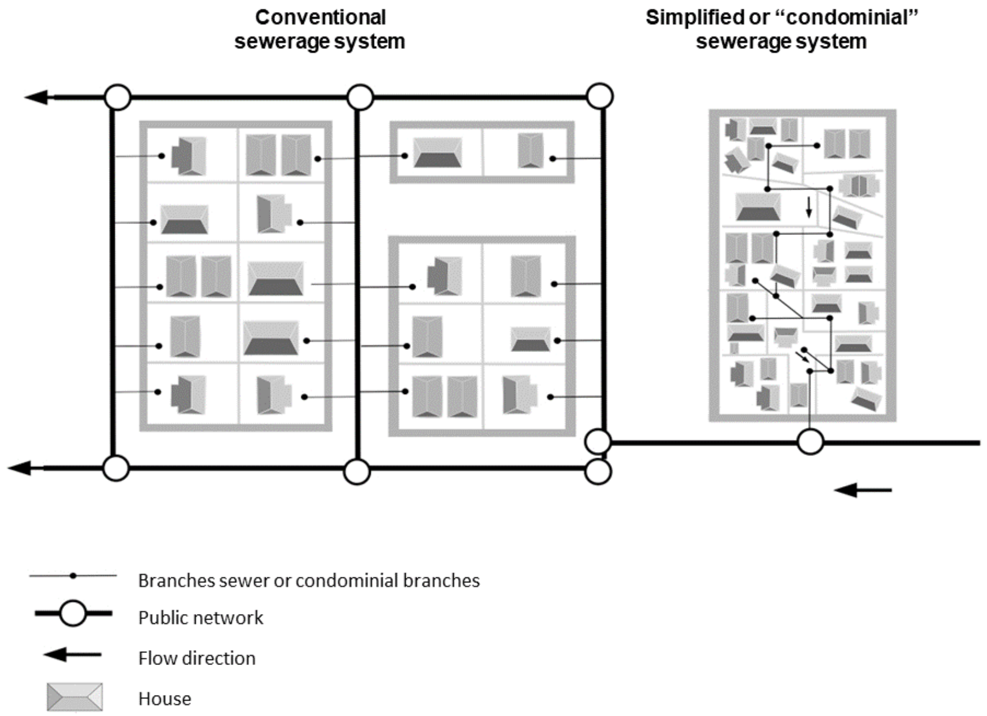

w only

BMJ Open

Simplified sewerage to prevent urban leptospirosis transmission: a cluster non-randomized controlled trial protocol in disadvantaged urban communities of Salvador, Brazil

Journal:	*BMJ Open*
Manuscript ID	bmjopen-2022-065009.R1
Article Type:	Protocol
Date Submitted by the Author:	24-Jan-2023
Complete List of Authors:	Cremonese, Cleber; Federal University of Bahia,<br>SOUZA, FABIO; Universidade Federal da Bahia<br>Palma, Fabiana ; Federal University of Bahia<br>Sodré, Jonatas Fernandes; Federal University of Bahia<br>Brito, Ricardo; Universidade Federal da Bahia<br>Ribeiro, Priscyla dos Santos ; Federal University of Bahia<br>Santana, Juliet ; Federal University of Bahia<br>Coelho, Rachel Helena; Federal University of Bahia<br>Ticona, Juan P. Aguilar ; Federal University of Bahia<br>Nazaré, Romero ; Fundação Oswaldo Cruz<br>de Oliveira, Daiana ; Federal University of Bahia<br>Silva, Cainã ; Federal University of Bahia<br>Eyre, Max ; Lancaster University<br>Mendes, Vinícius; Universidade Federal da Bahia<br>Knee, Jackie ; London School of Hygiene and Tropical Medicine Faculty of Infectious and Tropical Diseases<br>Ristow, Paula ; Federal University of Bahia<br>Stauber, Christine; Georgia State University<br>López, Yeimi Alexandra; Federal University of Bahia<br>Giorgi, Emanuele; Lancaster University Lancaster Medical School,<br>Diggle, Peter; Lancaster University, Faculty of Health and Medicine; University of Liverpool, Epidemiology and Population Health<br>Reis, Mitermayer Galvão ; Fundação Oswaldo Cruz<br>Cumming, Oliver; London School of Hygiene and Tropical Medicine, Department of Disease Control<br>Ko, Albert; Yale University,<br>Costa, Federico ; Federal University of Bahia
**Primary Subject Heading**:	Global health
Secondary Subject Heading:	Infectious diseases, Public health, Research methods
Keywords:	EPIDEMIOLOGY, Public health < INFECTIOUS DISEASES, BACTERIOLOGY

SCHOLARONE™
Manuscripts

Title: Simplified sewerage to prevent urban leptospirosis transmission: a cluster non-randomized controlled trial protocol in disadvantaged urban communities of Salvador, Brazil

Author affiliations

Cleber Cremonese[1], Fábio Neves Souza[1,2,3], Fabiana Almerinda Gonçalves Palma[1,2], Jonatas Fernandes Araújo Sodré[1], Ricardo Lustosa Brito[1], Priscyla dos Santos Ribeiro[4], Juliet Oliveira Santana[2], Rachel Helena Coelho[5], Juan P. Aguilar Ticona[1,2], Romero Nazaré[2], Daiana de Oliveira[1,2], Cainã Queiroz Silva[1], Max T. Eyre[6], Vinícius de Araújo Mendes[5] ,Jackie Knee[7], Paula Ristow[3], Christine Stauber[8], Yeimi Alexandra Alzate López[1], Emanuele Giorgi[6], Peter John Diggle[6], Mitermayer Galvão dos Reis[2,9], Oliver Cumming[7], Albert I. Ko[2,10] and Federico Costa[1,2,6,10]

[1]Institute of Collective Health, Federal University of Bahia, 40110-040, Salvador, Bahia, Brazil

[2]Instituto Gonçalo Moniz, Fundação Oswaldo Cruz, Ministério da Saúde, Rua Waldemar Falcão, 121, Salvador, Bahia, Brasil

[3]Institute of Biology, Federal University of Bahia, Ondina Campus, 40170-115, Salvador, Bahia, Brazil

[4]Institute of Biological Science, Federal University of Minas Gerais, Belo Horizonte, Brazil

[5]Faculty of Economics, Federal University of Bahia, Dois de Julho, Salvador, 40070-010, Salvador, Bahia, Brazil

[6]Lancaster Medical School, Lancaster University, Lancaster, LA1 4YW, UK

[7]London School of Hygiene & Tropical Medicine, Faculty of Infectious Tropical Diseases, Disease Control Department, United Kingdom

[8]Department of Population Health Sciences, School of Public Health, Georgia State University, Atlanta, GA, USA

For peer review only

[9]Faculty of Medicine of Bahia, Federal University of Bahia, Praça Conselheiro Almeida Couto, s/n - Largo do Terreiro de Jesus, Salvador, BA 40025-010, Salvador, Bahia, Brazil

[10]Department of Epidemiology of Microbial Diseases, School of Public Health, Yale University, 60 College Street, P.O. Box 208034, New Haven, CT, USA

Correspondence to: Cleber Cremonese (cleber.cremonese@gmail.com) and Federico Costa (federico.costa@ufba.br)

For peer review only

ABSTRACT

Introduction

Leptospirosis is a globally distributed zoonotic and environmentally mediated disease that has emerged as a major health problem in urban slums in developing countries. Its etiological agent is bacteria of the genus *Leptospira,* which are mainly spread in the urine of infected rodents, especially in an environment where adequate sanitation facilities are lacking, and it is known that open sewers are key transmission sources of the disease. Therefore, we aim to evaluate the effectiveness of a simplified sewerage intervention in reducing the risk of exposure to contaminated environments and *Leptospira* infection and to characterize the transmission mechanisms involved.

Methods and Analysis

This matched quasi-experimental study-design using non-randomized intervention and control clusters was designed to assess the effectiveness of an urban simplified sewerage intervention in the low-income communities of Salvador, Brazil. The intervention consists of household-level piped sewerage connections and community engagement and public involvement activities. A cohort of 1,400 adult participants will be recruited and grouped into eight clusters consisting of four matched intervention-control pairs with approximately 175 individuals in each cluster in baseline. The primary outcome is the seroincidence of *Leptospira* infection assessed through five serological measurements: one pre-intervention (baseline) and four post-intervention. As a secondary outcome, we will assess *Leptospira* load in soil, before and after the intervention. We will also assess *Leptospira* exposures before and after the intervention, through transmission modeling, accounting for residents' movement, contact with flooding, contaminated soil and water, and rat infestation, to examine whether and how routes of exposure for *Leptospira* change following the introduction of sanitation.

Ethics and dissemination

This study protocol has been reviewed and approved by the ethics boards at the Federal University of Bahia and the Brazilian National Research Ethics Committee. Results will be

For peer review only

disseminated through peer-reviewed publications and presentations to implementers, researchers, and participating communities.

Trial registration number: Brazilian Clinical Trials Registry, RCT trial RBR-8cjjpgm.

STRENGTHS AND LIMITATIONS OF THIS STUDY

- The first rigorous controlled intervention study for the effect of sanitation on Leptospira transmission the findings intend to guide future sanitation policies in communities with similar socio-economic status and environmental contexts.
- This is a cluster-based non-randomized controlled trial with intervention clusters receiving a simplified sanitation intervention and matched control clusters no intervention.
- The alliance between the natural and social sciences and the potential that it has to deepen community perceptions about leptospirosis and interventions before and after, as well as involvement in it in some decision-making processes in the improvement of their health, wellbeing, and sanitation.
- The allocation is non-randomized with selection based on intervention criteria determined by the implementer may have diluted intervention effects.
- Intervention will be carried out by the local public water and sanitation company, following their own planning, with no input or interference from the research team in the schedule or implementation.

INTRODUCTION

Globally, leptospirosis is a leading zoonotic disease in terms of morbidity and mortality [1], causing >1 million cases and >50,000 deaths yearly [1–3]. This zoonosis is an environmental and sanitation-related disease [4] characterized by annual epidemics during periods of increased rainfall and flooding [5,6]. Infection occurs frequently following exposure to water and soil that has been contaminated with the urine of infected animals [7,8]. Leptospirosis is an emerging disease in urban areas of most low- and middle-income countries (LMICs) [1], where a significant fraction of the population lives in disadvantaged urban communities (often called slums or *favelas* in Brazil) [9]. In those settings, poverty, precarious housing conditions, overpopulation, and lack of basic sanitation enable a high density of rats (the main urban reservoir hosts [10–13]) and high

For peer review only

88 leptospirosis transmission [14,15]. As the population living in urban disadvantaged communities will double to 2 billion by 2025 [16] the burden of leptospirosis is expected to increase.

90 Public health intervention strategies to prevent leptospirosis transmission are essential and must target the infrastructure deficiencies to prevent or diminish human exposure to environmental contamination sources. Governmental interventions focused on improving sanitation are critical for reducing the burden of leptospirosis as well as other diseases, like diarrheal [4,17]. However, these interventions, especially in disadvantaged urban communities, are not a priority or foreseeable soon considering the high cost of providing and maintaining such sanitation infrastructure. Exclusion of the disadvantaged urban communities' health issues in the government's political agenda [18–20], contributes to historical and structural disparities. Brazil, since the eighties, has been recognized as a global leader in the development of cost-effective water and sanitation systems for densely populated urban settings [21,22]. These systems, called simplified or "condominial" water and sewerage systems, are designed to be installed in densely populated, informal communities that are often situated in the kind of hilly terrain, common in Brazilian *favela* communities. The simplified sewerage approach is unique because it redefines the service unit as a community block rather than an individual household. It uses smaller pipes and inspection chambers (or cleanouts) at key junctions to facilitate installation and maintenance [21,22]. The system design reduces costs by decreasing the length of the trenches needed for sewerage lines and by using smaller pipes made from less expensive materials such as PVC (polyvinyl chloride). The installation of this alternative system can cost 50-70% less than a conventional system [21]. The lower costs associated with the installation of simplified systems makes these systems a more feasible alternative to conventional sewer systems in poor, resource-constrained settings. However, the simplified sewerage approach requires intensive community mobilization and engagement [23] as it is residents who take on leadership roles and have responsibility for decision-making.

113 Health impact evaluations of simplified sewerage approach in preventing disease has previously been limited to diarrheal diseases [24]. The evaluation of such interventions is challenging because of methodological problems such as cost and time of implementation, the requirement of large sample sizes, difficulties with randomization of the intervention allocation, and confounding factors (e.g., rodent infestation and Social-Economic Status). Contact with

For peer review only

environments contaminated with sewer overflow or residents' exposure to open sewerage near the household can influence the degree of individual exposure to *Leptospira* bacteria[14]. Humans exposure to *Leptospira* contamination through poor sanitation is mediated by individual behavior, socio-economic and environmental factors, rainfall, and flooding [4]. Therefore, the mechanisms of *Leptospira* transmission have different but important components of the environmental transmission route, for which their relative contribution is unknown because of confounding factors. Only studies that integrate a rigorous characterization of contamination sources (*Leptospira* load) and residents' movement could separate their effects and understand how source reduction interventions impact those transmission mechanisms. Determining the contribution of each of these mechanisms to *Leptospira* infection would help us to simultaneously understand the relationships and pathways that drive transmission [4]. This information is key to making interventions generalizable to other environmentally transmitted diseases that affect urban disadvantaged populations.

In this protocol, we describe an evaluation of a simplified sewerage intervention and community engagement with a quasi-experimental study-design using non-randomized intervention and control clusters. We aim to assess the longitudinal impact of a simplified sewerage intervention on the incidence and transmission mechanisms of *Leptospira* infection. In addition, we intend to investigate the impact of these interventions on reducing other water and environmental-related transmissible diseases.

Research aims and Hypotheses

We hypothesize that the combination of a simplified sewerage infrastructure and community engagement activities will reduce the risk of *Leptospira* infection among disadvantaged urban communities in Salvador, Brazil. We further hypothesize that this approach will be effective in reducing: 1) frequency of human contact with open sewerage contaminated with pathogenic *Leptospira*; 2) rat infestation; and 3) *Leptospira* load in the soil around the simplified sewerage**.** We aim to evaluate the efficacy of a sewerage intervention on reducing *Leptospira* infection and other health outcomes related to sanitation in disadvantaged urban communities in Brazil. Our specific objectives are to: (1) to evaluate prospectively the effectiveness of a simplified sewerage intervention in reducing the risk of *Leptospira* infection; (2) to determine the mechanisms by which the simplified sewerage reduce direct human contact with

For peer review only

environmental pathogen load in urban slums. **Figure 1** provides further details on our hypotheses concerning the effects of the sanitation intervention on the transmission mechanisms of urban leptospirosis. We conceptualize this protocol as focusing on the sanitation intervention to decrease leptospirosis; however, this study protocol generates rich opportunities to evaluate other health outcomes. Therefore, we additionally aim to investigate the effects of the sewerage intervention on childhood exposure to a range of enteropathogens, and long-term child neurodevelopment and anthropometry. As exploratory outcomes, we will also investigate the effect of the sewerage intervention on the reduction of risk of arbovirus infectious diseases.

METHODS AND ANALYSIS

Overview of study design

This is an intervention trial with a cluster non-randomised controlled trial, with eight clusters (four intervention and four control), including longitudinal serological follow-up of 1,400 adults' residents from disadvantaged urban communities at high risk for leptospirosis transmission in Salvador, Bahia, Brazil. The primary outcome will be *Leptospira* infection. Each pair of intervention and control clusters will be allocated in distinct hydrological sub-basins with distances from 1,000 to 1,500 meters apart. Clusters will be matched on socio-economic status (SES), topographic or hydrologic characteristics, population density, and *Leptospira* seroprevalence. The cluster selection will also consider exposure to the pathogen by selecting houses that are located to similar distances to new sewerage system and control open sewers. The intervention aims at a combination of sanitation (simplified sewerage intervention) and community engagement activities.

Study settings

Salvador is the third largest city in Brazil with a population of 2.9 million residents [25]. Using a surveillance database of active leptospirosis in the study setting between 1996 and 2018, we performed a population-based, adjusted Kernel analysis among geo-localized records of households with leptospirosis cases to identify high risk area corresponding to 8.06% of the total area of the city [15,26]. We selected eight clusters (four intervention and four control) with average size 0.03 Km^2 located in these areas. The intervention clusters will be located in areas where the

For peer review only

provision of simplified sewerage and community engagement is planned. Moreover, the choice of the control group is dependent on the implementer's information regarding non-intervention planned in the area for a short-term period of 5 years. Geographic location of intervention and control clusters is detailed in **Figure 2**.

Description of the Sewerage intervention

During 2022 and 2023, the sewerage intervention (simplified sewerage intervention) will be implemented by the Public Water and Sanitation Bahia State Company (*Empresa Baiana de Águas e Saneamento*) in different urban locations in Salvador, Brazil. The expansion of the sanitation coverage in the city has generated an opportunity to assess the longitudinal effectiveness of the intervention on the transmission of sanitation-related diseases, such as leptospirosis. The company is government-owned, with responsibilities for managing, monitoring, and distributing water and sanitation services throughout the state of Bahia. The costs, management, and entire execution of the work will be the responsibility of the water and sanitation company, while the team of researchers will only monitor the development of the work.

This simplified sewerage intervention is characterized by receiving the sewage generated by a cluster of houses instead of individual houses. In conventional sewerage system designs, each household is connected directly to a principal line while in the sewer simplified system households are connected to branch lines within each block [24] **(Supplementary figure 1)**.

The sanitation company will carry out the sewerage network works, which include the installation of a 150 mm primary pipe at the bottom of the residential lot, bordering a watercourse. Household sewerage networks are interconnected on one main line connecting to the main network, thus the intervention is expected to increase sanitation coverage in the study clusters by expanding the collection network. These interventions would exclusively be of a sewerage nature, with no complementary functions such as local drainage. Importantly, the intervention also includes community participation in the action designs and implementation.

Community engagement

Simplified sewerage includes different degrees of social participation. We recognize that the intervention in sanitary sewerage impacts the local environment and the communities' perception

For peer review only

is affected by the sanitation interventions. Problems and failures related to sewerage intervention have been reported as mainly due to the difficulties in the relationship between the technicians who accompany the works and the communities which reduced the community participation to sewerage maintenance [23].

Faced with this complexity related to urban sanitation, the community engagement processes that will be implemented in this study are focused on approaches that would stimulate community mobilization to participate in the research processes, popular health education actions, and in the evaluation of the implementation of sewerage intervention. Popular Health Education (PHE) actions will be oriented toward disease prevention and health promotion of the population. The PHE is a "set of practices targeted at individuals and the community" [27,28], and this study intends to contribute to community empowerment and support for the management of sewerage intervention. The community engagement activities would be implemented in stages and with different stakeholders, including the community leaders, residents, and a multidisciplinary team of researchers including epidemiologists, ecologists, sanitation engineers, anthropologists, environmental microbiologists, geographers, economists, statisticians, etc. The study, therefore, aims to strengthen community participation in the decision-making in the management of sewerage intervention, and also support the involvement of residents in activities research such as (1) Community and institutional mobilization; (2) Collaborative mapping; and (3) Popular health education actions. Moreover, we will perform community ethnography to deepen the understanding of the social and cultural impacts caused before and after the intervention, as well as its environmental impacts and the mobilization processes.

Study population and sample size

To evaluate the effectiveness of the simplified sewerage intervention in reducing the risk of *Leptospira* infection, we plan to recruit an estimated 1,400 participants aged ≥18 years, residing in the selected clusters (approximately 175 adults per cluster in baseline). To evaluate the intervention effect on secondary (sewerage contact, environmental pathogen load, long-term child development) and exploratory (arbovirus infections, diarrheal disease, and enteric infection) outcomes, we plan to increase our sample size by 420 (30%) participants, aged between 6 months and 17 years, totaling 1,840 people. All participants from both intervention and control

For peer review only

clusters will be followed over a period of three years after the sewerage intervention and a baseline prior to the intervention **(Figure 2)**. In the flow diagram, we provide a timeline and trial study phases in detail (**Figure 3)**.

The sample size was estimated using data on *Leptospira* infection from a previous leptospirosis cohort study in Salvador, Brazil [14]. We divided the previous leptospirosis cohort study [14] into areas of similar size to that the clusters used trial and calculated the between-cluster variation. Of 1,840 individuals participating in the previous cohort, 142 (7.9%) experienced leptospiral infection (k/ICC=0.03). Power calculations are based on a cluster-level using effect sizes (% decrease in incidence between intervention and control clusters) 40, 50, and 60%, assuming an infection rate of 0.08 per person-year. All sample size calculations used methods for matched cluster-randomized trials with the study structure of four matched pairs of clusters. Sample size estimates assumed a 12% annual drop-out rate across the three-year study period.

Control selection

Control clusters (CONT) will be selected in locations with an incomplete sewerage sanitation system. Matching criteria for control clusters include: location in a geographic area that has similar environmental (elevation, hydrologic data) and social (income, education, population density) characteristics to the intervention clusters; and at a distance of 1,000 and 1,500 meters from the intervention cluster. Areas that received major infrastructure interventions and sanitation improvements in the last five years will be excluded from the selection frame for control clusters.

Eligibility and enrolment

Before the recruitment of study participants, a census will be carried out within the selected eight clusters to enumerate all residents from households within <40m distance from an open sewer. This would ensure that households and residents living in areas with a higher risk of leptospirosis transmission are included. At each census, the individual data (name, sex, age) and the geo-coordinates a primary household respondent would be collected. As a further step, we will randomly select population-based cohorts with 95 households (175 participants) per cluster. Residents will be eligible for inclusion in the study, if (1) they are 18 years of age or above, (2) have lived in the area for at least six months, (3) sleep in the household for at least 3 nights per week, and (4) consent to the study procedures and complete an informed consent form. However,

For peer review only

residents will be excluded if: (1) they do not meet all of the inclusion criteria; (2) they cannot provide written informed consent to participate in the study, (3) they refuse or are unable to provide demographic information and biological sample; (4) they are with limited mental health problems or other problems that have implications for survey responses or participation in any other phase of research activity.

For secondary outcomes we will include all children and young between 6 months and 17 years of age will be included in the study **(Supplementary table 1)**. We will apply similar inclusion and exclusion criteria as described but excluding children for which their legal guardians do not provide written informed consent for their participation.

Outcomes

Primary outcome measure

The study trial will consist of three specifics phases: (1) baseline; (2) sanitation intervention implementation; (3) follow-up of the residents and evaluation of the sanitation intervention **(Figure 2)**. During the phase (1) a baseline survey will be conducted to characterize the demographics, socio-economic status and *Leptospira* seroprevalence in each cluster. During the phase (3) biannual serosurveys in the cohorts will be conducted to measure the primary outcome, *Leptospira* asymptomatic infection, and estimate the risk of infection during the dry and rainy seasons. The field team will collect blood samples from eligible participants with age ≥18 years. A blood sample (10mL) will be collected from each study participant and examined for *Leptospira* antigen using the microscopic agglutination test diagnostic (MAT) [4]. MAT is the standard test to determine *Leptospira* infection in longitudinal studies and *Leptospira* infection will be defined as a four-fold rise in MAT titer or seroconversion (negative to ≥1:50) between samples from consecutive serosurveys. We will use a panel of six reference strains: representing *L. interrogans* serovars Autumnalis strain Akiyami A, Canicola strain H. Utrecht IV and Icterohaemorrhagiae strain RGA, *L. borgpetersenii* serovar Ballum strain MUS 127, *L. kirschneri* serovar Cynopteri strain 3522C and Grippothyphosa strain Duysterand the two clinical isolates: *L. interrogans* serovar Copenhageni strain Fiocruz L1-130 (isolated locally in 1996) and *L. santarosai* serogroup Shermani strain LV3954 (isolated locally). This procedure had the same performance in identifying MAT seroconversion in our prospective studies [4,15] compared with the WHO

For peer review only - http://bmjopen.bmj.com/site/about/guidelines.xhtml

recommended panel of 19 reference serovars. The screening will be performed with serum dilutions of 1:50-1:100. When agglutination is observed at 1:100, the sample will be titrated to determine the highest titer. As part of quality control procedures, two independent evaluations will be conducted for 8% of the samples and all samples from infected subjects.

Secondary outcomes

Environmental exposure secondary outcomes

Secondary outcomes for environmental effects will include microbiological assays to assess soil contamination with pathogenic *Leptospira*. The presence and concentration of pathogenic *Leptospira* will be evaluated in the eight clusters. Bases on prevalence the contamination soil with *Leptospira* in studies previous and packing density of 0.4 with minimum distances between collection points, a total of 68 soil samples and 15 water sewage samples will be collected in each cluster and transported refrigerated to the laboratory within six hours for processing. Sampling will take place in duplicate, with intervals of seven to nine days between them. After processing, soil and sewage samples will be stored at -80°C until molecular assays can be performed. The presence and concentration of pathogenic *Leptospira* will be evaluated using a qPCR assay (quantitative polymerase chain reaction), targeting the *lipL32* gene. Samples will be considered positive when the two qPCR replicates show amplification before a Cq of 40 [8]. Environmental variables for rainfall, temperature, landscape (remote sensing using drones), flooding, and hydrological features will also be collected during rainy seasons before and at least three months immediately after the sewerage intervention.

Rats Survey

Rat abundance will be examined at each cluster using tracking boards to quantify the level of rat activity, before and after the interventions. The use of tracking boards to quantify rat presence and activity (as rat abundance proxy) will be performed, following previously described, validated, and optimized methods [29]. Tracking boards will be distributed in each cluster for two days in a row and will be scored daily. The overall level of rat presence for each cluster will be estimated based on the percentage of positive tracking boards and the intensity of marks on each board before and at least three months immediately after the sewerage intervention.

For peer review only

Human movement

Environmental exposure to *Leptospira* (i.e., proximity to open sewer and unpaved roads; housing conditions; and flooding) will be quantified by studying of the individual movement of the participants in each study cluster. Movement evaluations will be performed biannually, before and after the sewerage intervention. We will focus on a sub-cohort of 320 residents living in each of the eight study clusters, divided into four groups: young males (18–34 years), young females (18–34 years), old males (aged ≥35 years), and old females (aged ≥35 years). We will use GPS data loggers that can measure small-scale movement patterns [30,31].

Forty residents in each of the study clusters will be invited to use a GPS data logger IgotU® GT-600 device (Mobile Action Technology, New Taipei City, Taiwan) [32]. Additionally, we will obtain information about movement and behavior through resident diaries and retrospective mapping before and after the intervention. Diaries will provide qualitative information on the number of hours inside/outside the study cluster and the household; contact duration and nature of the contact (i.e., contact duration with and without use of boots) with contaminated sources; and the activity performed (i.e., working, leisure, etc.) for 7 days. Retrospective mapping will provide information on the past 24 hours' pathways traveled by the residents, and contacts with contamination sources from the residents' perspective [31]. To determine participants' compliance with the study protocol using the GPS data logger and diary entry is defined as adherence for blood collection and direct observations will be conducted for participants ≥18 years of age. Non-compliance is defined as non-adherence to all study steps.

Other outcomes

Human health and wellbeing

The data will be collected pre and post-intervention to evaluate the impact on well-being between arms. Other outcomes related to individual and household health and well-being include subjective measures - self-reported general, physical and emotional well-being using the Short-Form Health Survey-12 (SF-12) [33]; perceived living conditions questions; the short version (five items) of the Brazilian Food Insecurity Scale [34] which measures the perception and experience of hunger over the past 3 months; and objectives measures - productivity through employment

For peer review only

and income. We will also evaluate whether there will be a decrease in material and economic loss measures due to floods and landslides -which often occur in the areas without adequate sanitation.

Long-term child development

Enteric pathogens associated with diarrhea can inhibit a child's ability to absorb nutrients, further exacerbating malnutrition and developmental delays. Studies are indicating a growing association between enteric infections and environmental enteropathy, a disorder characterized by reduced intestinal barrier function, abnormal intestinal morphology, and increased inflammation [35,36]. This disorder is thought to stem from unsanitary conditions that lead to repeated exposures to enteric pathogens. Additionally, poor gut health, chronic immune stimulation and malnutrition, are linked to poor neurodevelopment in children, as well as stunting. In poverty-stricken environments, these nutritional and environmental risks can also affect the mother-child behavior patterns, which are critical for normal development, including a balance between time spent with caring their children and sanitation home issues [37].

Child neurodevelopment will be evaluated using the Ages & Stages Questionnaires 3rd edition (ASQ-3) for children aged 6 to 66 months and with the Brazilian National Assessment of School Performance (ANRESC - *Avaliação Nacional do Rendimento Escolar)* for children older than 66 months, in the pre- and post-intervention period. ASQ-3 is a tool used to monitor the child's developmental progress, it includes fine and gross motor, communication, problem-solving, and personal-social domains [38]. ANRESC is part of the Brazilian Basic Education Assessment System, which included the assessment of Math and Language skills [39]. The child development assessment will also include measurements of weight, height, BMI and head circumference. We will use the WHO Child Growth Standards and the results will be summarized using the Z-score [40].

Enteric diseases

To evaluate the impact of the intervention on enteric pathogen exposure among young children and, we will collect stool and/or rectal swab samples from children aged 6 months to 5 years old in the pre-post-intervention periods. We will use a custom-designed TaqMan Array Card (TAC) to

For peer review only

analyse samples for the presence of over 30 enteric pathogens, including viruses, bacteria, protozoa, and helminths. The TAC uses microfluidic technology to simultaneously perform 48 individual quantitative polymerase chain reactions per sample and has been previously validated and used for detection enteric pathogens in stool [41–43].

Arboviral diseases

We will evaluate the effectiveness of the sewerage intervention and the changes in landscape characteristics associated with them, in the spatiotemporal distribution of arbovirus (Dengue, Zika, Chikungunya) vector mosquitoes. We will carry out entomological investigations in private (participants' homes) and public areas with the collection of adult and immature mosquitoes by aspiration [44], with the verification of identified mosquitoes breeding sites, to measure their density. We will verify whether there is a spatiotemporal pattern in the presence and density of vector mosquitoes in the sampled regions, to allow us to investigate the possible factors that may be related to changes in the distribution of exposures before, during, and after the intervention process and whether these interventions have modified ecological indices for vector abundance.

Data Collection and management

Trained interviewers will administer specific questionnaires to participants through face-to-face interviews to collect general demographic information, socio-economic data, exposure factors and individual or collective sanitation practices, and knowledge about leptospirosis and other diseases. In the case of children at home, a new questionnaire will be answered by the caregiver. Information will be collected on the child's general health status, development, and medical history and diseases **(Supplementary table 2)**. The questionnaires and all information will be confidential and coded using individually identifiable numeric identifiers. Every effort will be made to protect the privacy of the study participants and all data collected will be treated as confidential.

All questioners and forms described in this protocol will be included in the Research Electronic Data Capture (REDCap) database. The REDCap web version as well as its mobile application will be used to record all data using mobile devices. A data management staff will keep track of the information in real time.

Participants and public involvement

For peer review only

All study subjects will be involved in the trial, answering questionnaires, providing biological samples, and involved in engagement activities as previously described in the section on community engagement. The sanitation intervention will be conducted by a governmental agency, and the implementation of this intervention involves dialogue with the residents of the communities. However, the study design and conduct of the trial of this intervention study will be taking place independently of public involvement. After each follow-up completion, research findings will be shared and disseminated in a group's meetings or visit households with key stakeholders.

Statistical analysis

Descriptive statistics will be employed to report the demographic, social, economic, or environmental characteristics between the study clusters (intervention and control clusters). For the primary outcome, we will apply quasi-Poisson regression to estimate the incidence rate ratio (IRR) of *Leptospira* infection. We will also evaluate the effect of the intervention on *Leptospira* infection risk using mixed-effects logistic regression models to account for clustering of the observations within study units and across repeated measures for individuals. We will model the overall effect of the intervention on infection risk while controlling for baseline sewerage and flooding exposure and potential confounding variables, such as age, sex, indicators of the socio-economic level, and rainfall intensity during an annual period, which are presumably unrelated to the intervention **(Figure 1)**.

For the secondary outcome, we will conduct environmental analyses of *Leptospira* load between intervention and control clusters to compare environmental risk. To do this, we will model two leptospiral load outcomes, prevalence (binomial) and concentration (log-transformed), using generalized linear models with random effects to account for clustering within study units. The association between environmental contamination and hypothesized predictors of risk (rat abundance, human movement, and ecological data) will also be explored in the model **(Figure 1)**.

Limitations and bias

The allocation is non-randomised with selection based on intervention criteria determined by the implementer so the risk of residual confounding cannot be excluded. The choice of the control

For peer review only

group is also dependent on the implementer's information about the non-intervention in the area in the short term (5 years-period). To limit the potential for bias arising from our non-randomised design, we will select control group with as similar as possible socio-economic and environmental characteristics to those of their matched intervention clusters.

We will perform the impact calculation through the double differentiation of the averages of the results found between the intervention and control clusters before and after the sewerage intervention. The mitigation of bias will be carried out through the control of fixed effects of time and location between different intervention and control clusters over the years of evaluation.

ETHICS AND DISSEMINATION

This project was evaluated and approved by a local ethics committee (CEP/ISC/UFBA) under number CAEE 32361820.7.0000.5030, and a national research ethics committee (CONEP) linked to the Brazilian Ministry of the Health under approval number 4.235.251. The trial study protocol was registered with the Brazilian Clinical Trials Registry, RCT trial RBR-8cjjpgm (https://ensaiosclinicos.gov.br/rg/RBR-8cjjpgm). All participants involved in the study will provide informed consent before any study activity, and the community leaders, previously identified by the team, will provide verbal consent before enrollment of the community in the trial. Participants aged 12 and 17 will sign an assent form, in addition to a participation consent form completed through their parents or legal guardians. Children under the age of 12 will have their consent signed by their parents or legal guardians only. The results of the trial will be presented at local and international meetings and submitted to peer-reviewed journals for publication. Results will also be shared directly with the participating communities. All individual information participants will be kept and published anonymously.

Authors' contributions

FC, CC, MGR, AK, YAL, PD, CS and OC contributed with project's main conceptual ideas; FC, ME, EG, and CC contributed to study design and statistical analysis plan; FNS, DO, FAP, CS, JFA, PSR, JOS, RHC, DS, RN, RLB, VAM, JPAT, JK, PR, FC, and CC developed field and laboratory protocols and implementation; FNS, FAP, CC, RHC, and FC developed data collection

For peer review only

tools; FNS, FAP, JFA, RHC, PR, CC, and FC contributed to drafting the main manuscript. All authors contributed to editing and revising the manuscript.

Funding statement

This work was supported by Wellcome Trust grant number 102330/Z/13/Z. All project costs are jointly funded by Wellcome Trust and the Department of Health and Social Assistance (DHSC), through the National Institute for Health Research (NIHR), using the UK Official Development Assistance Fund (ODA).

Competing interest statement

Not applicable

Data sharing statement

The data collected with personal identifying data redacted will be available encoded for use by principal investigators on request following the publication of trial results. The funders have no role in the study design, data collection, and analysis, decision to publish, or preparation of manuscripts.

Acknowledgments

The authors gratefully acknowledge critical input on the study protocol and all the study participants. We acknowledge the partner institutions for their collaboration and dedication. We thank Patricia Lustosa, and Mayara Carvalho for their help with the review and discussion of this protocol, Ana Maria Silva and George G. Machado for help with supplementary figure, and Hammed O. Mogaji for his help with manuscript proofing.

REFERENCES

1. Costa F, Hagan JE, Calcagno J, Kane M, Torgerson P, Martinez-Silveira MS, et al. Global Morbidity and Mortality of Leptospirosis: A Systematic Review. PLoS Negl Trop Dis. 2015;9: e0003898.
2. Ko AI, Goarant C, Picardeau M. Leptospira: the dawn of the molecular genetics era for an emerging zoonotic pathogen. Nat Rev Microbiol. 2009;7: 736–747.

For peer review only

3. Bharti AR, Nally JE, Ricaldi JN, Matthias MA, Diaz MM, Lovett MA, et al. Leptospirosis: a zoonotic disease of global importance. Lancet Infect Dis. 2003;3: 757–771.

4. Khalil H, Santana R, de Oliveira D, Palma F, Lustosa R, Eyre MT, et al. Poverty, sanitation, and Leptospira transmission pathways in residents from four Brazilian slums. PLoS Negl Trop Dis. 2021;15: e0009256.

5. Ko AI, M GR, Cm RD, Johnson WD, Riley LW. Urban epidemic of severe leptospirosis in Brazil. Salvador Leptospirosis Study Group. Lancet. 1999;354. doi:10.1016/s0140-6736(99)80012-9

6. Sarkar U, Nascimento SF, Barbosa R, Martins R, Nuevo H, Kalofonos I, et al. Population-based case-control investigation of risk factors for leptospirosis during an urban epidemic. Am J Trop Med Hyg. 2002;66. doi:10.4269/ajtmh.2002.66.605

7. Bharti AR, Nally JE, Ricaldi JN, Matthias MA, Diaz MM, Lovett MA, et al. Leptospirosis: a zoonotic disease of global importance. Lancet Infect Dis. 2003;3: 757–771.

8. Casanovas-Massana A, Pedra GG, Wunder EA Jr, Diggle PJ, Begon M, Ko AI. Quantification of Leptospira interrogans Survival in Soil and Water Microcosms. Appl Environ Microbiol. 2018;84. doi:10.1128/AEM.00507-18

9. Programme UNHS, United Nations Human Settlements Programme. World Cities Report 2020. World Cities Report. 2020. doi:10.18356/27bc31a5-en

10. Minter A, Diggle PJ, Costa F, Childs J, Ko AI, Begon M. Evidence of multiple intraspecific transmission routes for Leptospira acquisition in Norway rats (Rattus norvegicus). Epidemiol Infect. 2017;145: 3438–3448.

11. Panti-May JA, Carvalho-Pereira TSA, Serrano S, Pedra GG, Taylor J, Pertile AC, et al. A Two-Year Ecological Study of Norway Rats (Rattus norvegicus) in a Brazilian Urban Slum. PLoS One. 2016;11: e0152511.

12. Costa F, Wunder EA Jr, De Oliveira D, Bisht V, Rodrigues G, Reis MG, et al. Patterns in Leptospira Shedding in Norway Rats (Rattus norvegicus) from Brazilian Slum Communities at High Risk of Disease Transmission. PLoS Negl Trop Dis. 2015;9: e0003819.

13. Costa F, Porter FH, Rodrigues G, Farias H, de Faria MT, Wunder EA, et al. Infections by Leptospira interrogans, Seoul virus, and Bartonella spp. among Norway rats (Rattus norvegicus) from the urban slum environment in Brazil. Vector Borne Zoonotic Dis. 2014;14: 33–40.

14. Hagan JE, Moraga P, Costa F, Capian N, Ribeiro GS, Wunder EA Jr, et al. Spatiotemporal Determinants of Urban Leptospirosis Transmission: Four-Year Prospective Cohort Study of Slum Residents in Brazil. PLoS Negl Trop Dis. 2016;10: e0004275.

15. Reis RB, Ribeiro GS, Felzemburgh RDM, Santana FS, Mohr S, Melendez AXTO, et al. Impact of environment and social gradient on Leptospira infection in urban slums. PLoS Negl Trop Dis. 2008;2: e228.

16. United Nations Human Settlements Programme. The Challenge of Slums: Global Report on Human Settlements, 2003. UN-HABITAT; 2003.

17. Barreto ML, Genser B, Strina A, Teixeira MG, Assis AMO, Rego RF, et al. Effect of city-wide sanitation programme on reduction in rate of childhood diarrhoea in northeast Brazil: assessment by two cohort studies. Lancet. 2007;370: 1622–1628.

18. Bolay J-C. Slums and Urban Development: Questions on Society and Globalisation. The European Journal of Development Research. 2006;18: 284–298.

19. Staff UNHSP, Programme UNHS. The challenge of slums: global report on human settlements, 2003. UN-HABITAT; 2003.

For peer review only

20. IBGE – Instituto Brasileiro de Geografia e Estatística. In: Censo Demográfico 2020 [Internet]. [cited 23 Dec 2021]. Available: https://www.ibge.gov.br/

21. Melo JC. The experience of condominial water and sewerage systems in Brazil: case studies from Brasilia, Salvador and Parauebas. The World Bank; 2005.

22. Melo JC. Sistema Condominial: uma resposta ao desafio da universalização do saneamento. Ministério das Cidades. 2008.

23. Nance E, Ortolano L. Community Participation in Urban Sanitation: Experiences in Northeastern Brazil. Journal of Planning Education and Research. 2007;26: 284–300.

24. Hubbard B, Sarisky J, Gelting R, Baffigo V, Seminario R, Centurion C. A community demand-driven approach toward sustainable water and sanitation infrastructure development. Int J Hyg Environ Health. 2011;214: 326–334.

25. IBGE. Estimated Population 2017. Cities, Bahía, Salvador. [cited 11 Apr 2022]. Available: https://cidades.ibge.gov.br/brasil/ba/salvador/panorama

26. Srihera R, Stute W. Kernel adjusted density estimation. Stat Probab Lett. 2011;81: 571–579.

27. Seabra CAM, Xavier SPL, Sampaio YPC, de Oliveira MF, da Silva Quirino G, de Fátima Antero Sousa Machado M. Health education as a strategy for the promotion of the health of the elderly: an integrative review. Revista Brasileira de Geriatria e Gerontologia. 2019. doi:10.1590/1981-22562019022.190022

28. Falkenberg MB, Mendes T de PL, Moraes EP de, Souza EM de. Educação em saúde e educação na saúde: conceitos e implicações para a saúde coletiva. Ciênc saúde coletiva. 2014;19: 847–852.

29. Hacker KP, Minter A, Begon M, Diggle PJ, Serrano S, Reis MG, et al. A Comparative Assessment of Track Plates to Quantify Fine Scale Variations in the Relative Abundance of Norway Rats in Urban Slums. Urban Ecosyst. 2016;19: 561–575.

30. Vazquez-Prokopec GM, Stoddard ST, Paz-Soldan V, Morrison AC, Elder JP, Kochel TJ, et al. Usefulness of commercially available GPS data-loggers for tracking human movement and exposure to dengue virus. International Journal of Health Geographics. 2009. p. 68. doi:10.1186/1476-072x-8-68

31. Owers KA, Odetunde J, de Matos RB, Sacramento G, Carvalho M, Nery N Jr, et al. Fine-scale GPS tracking to quantify human movement patterns and exposure to leptospires in the urban slum environment. PLoS Negl Trop Dis. 2018;12: e0006752.

32. Morris G, Conner LM. Assessment of accuracy, fix success rate, and use of estimated horizontal position error (EHPE) to filter inaccurate data collected by a common commercially available GPS logger. PLoS One. 2017;12: e0189020.

33. Ware J Jr, Kosinski M, Keller SD. A 12-Item Short-Form Health Survey: construction of scales and preliminary tests of reliability and validity. Med Care. 1996;34: 220–233.

34. Santos LPD, Lindemann IL, Motta JVDS, Mintem G, Bender E, Gigante DP. Proposal of a short-form version of the Brazilian food insecurity scale. Rev Saude Publica. 2014;48: 783–789.

35. Donowitz JR, Drew J, Taniuchi M, Platts-Mills JA, Alam M, Ferdous T, et al. Diarrheal Pathogens Associated With Growth and Neurodevelopment. Clin Infect Dis. 2021;73: e683.

36. Website. Available: https://nyaspubs.onlinelibrary.wiley.com/doi/10.1111/nyas.12330

37. George CM, Perin J, Parvin T, Bhuyian MSI, Thomas ED, Monira S, et al. Diarrhea Prevalence and Child Growth Faltering Are Associated with Subsequent Adverse Child Developmental Outcomes in Bangladesh (CHoBI7 Program). Am J Trop Med Hyg.

For peer review only

2022;106: 233.

38. Filgueiras A, Pires P, Maissonette S, Landeira-Fernandez J. Psychometric properties of the Brazilian-adapted version of the Ages and Stages Questionnaire in public child daycare centers. Early Hum Dev. 2013;89: 561–576.

39. Brasil M, da Aneb IM. da Anresc 2015. Brasília: Inep. 2017.

40. de Onis M, Garza C, Victora CG, Onyango AW, Frongillo EA, Martines J. The WHO Multicentre Growth Reference Study: planning, study design, and methodology. Food Nutr Bull. 2004;25: S15–26.

41. Platts-Mills JA, Liu J, Rogawski ET, Kabir F, Lertsethtakarn P, Siguas M, et al. Use of quantitative molecular diagnostic methods to assess the aetiology, burden, and clinical characteristics of diarrhoea in children in low-resource settings: a reanalysis of the MAL-ED cohort study. The Lancet Global Health. 2018;6: e1309–e1318.

42. Liu J, Platts-Mills JA, Juma J, Kabir F, Nkeze J, Okoi C, et al. Use of quantitative molecular diagnostic methods to identify causes of diarrhoea in children: a reanalysis of the GEMS case-control study. Lancet. 2016;388: 1291–1301.

43. Liu J, Gratz J, Amour C, Nshama R, Walongo T, Maro A, et al. Optimization of Quantitative PCR Methods for Enteropathogen Detection. PLoS One. 2016;11: e0158199.

44. Vazquez-Prokopec GM, Galvin WA. A new, cost-effective, battery-powered aspirator for adult mosquito collections. Journal of medical. 2009. Available: https://academic.oup.com/jme/article-abstract/46/6/1256/921254

For peer review only

For peer review only - http://bmjopen.bmj.com/site/about/guidelines.xhtml

FIGURES

Figure 1- Rationale, hypothesis, and outcomes assessed in the study. We hypothesize that sanitation intervention will improve the environmental conditions and change the behavior of humans, resulting in both direct and indirect impacts on the incidence of leptospirosis and other diseases human and well-being. * HoH- Household-level

Figure 2- Study clusters in the city of Salvador, Brazil. Altitude and sampling clusters (**A**). Example of baseline sanitation conditions of the study cluster (**B**). In (**C**) Consort flow diagram by intervention and control cluster, data collection times and type of activities.

Figure 3- Overview of the study procedures, the timeline for study, and data collection. **Note 1**: Each cluster of the study will be formed by a cluster with intervention vs. Control. The period of activities for each serological survey will take place at the time described in the figure (Allocation, Recruitment, and Baseline). **Note 2**: Update years 1 to 5 will be realized after receipt of the water and sanitation company schedule. SES: Socio-economic status.

For peer review only

For peer review only

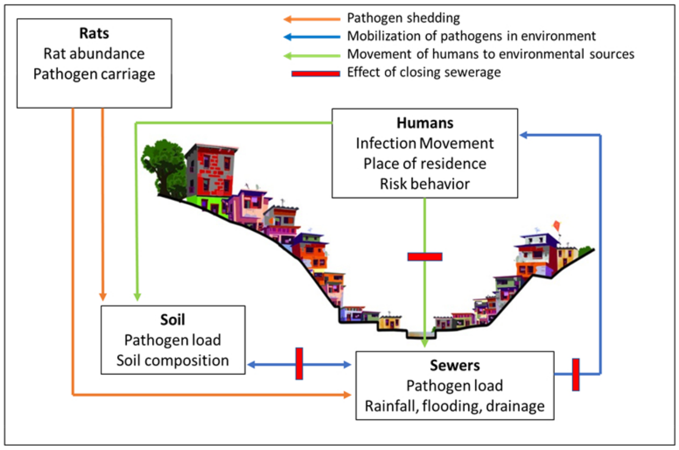

Measurements	
**Ascertainment**	**Measure**
**Cluster-level**	
Nat. Meteorol. Serv	Weather
Remote sensing survey	Land classification
GIS survey	Topology
	Hydrology, flood-risk
Environmental surveys	Open drainage
	Open refuse deposits
Intervention Company Database	Sewerage connections
Tracking board surveys	Rat abundance
Soil *lip*L32 qPCR survey	*Leptospira* DNA load
**Household-level**	
Interviews of HoH	Socio-economic Status
Environmental surveys	Informal sewerage
	Sewage line condition
Rodent infestation survey	Infestation score
Census	Domestic animals
**Individual-level**	
Engagement Communitarian	Community participation level
Interviews	Demographics
	Risk behaviours
	Time in cluster
Serosurveys	*Leptospira* infection
GPS tracking survey	Time in proximity to sources of contamination

Figure 1- Rationale, hypothesis, and outcomes assessed in the study. We hypothesize that sanitation intervention will improve the environmental conditions and change the behavior of humans, resulting in both direct and indirect impacts on the incidence of leptospirosis and other diseases human and well-being.

242x108mm (120 x 120 DPI)

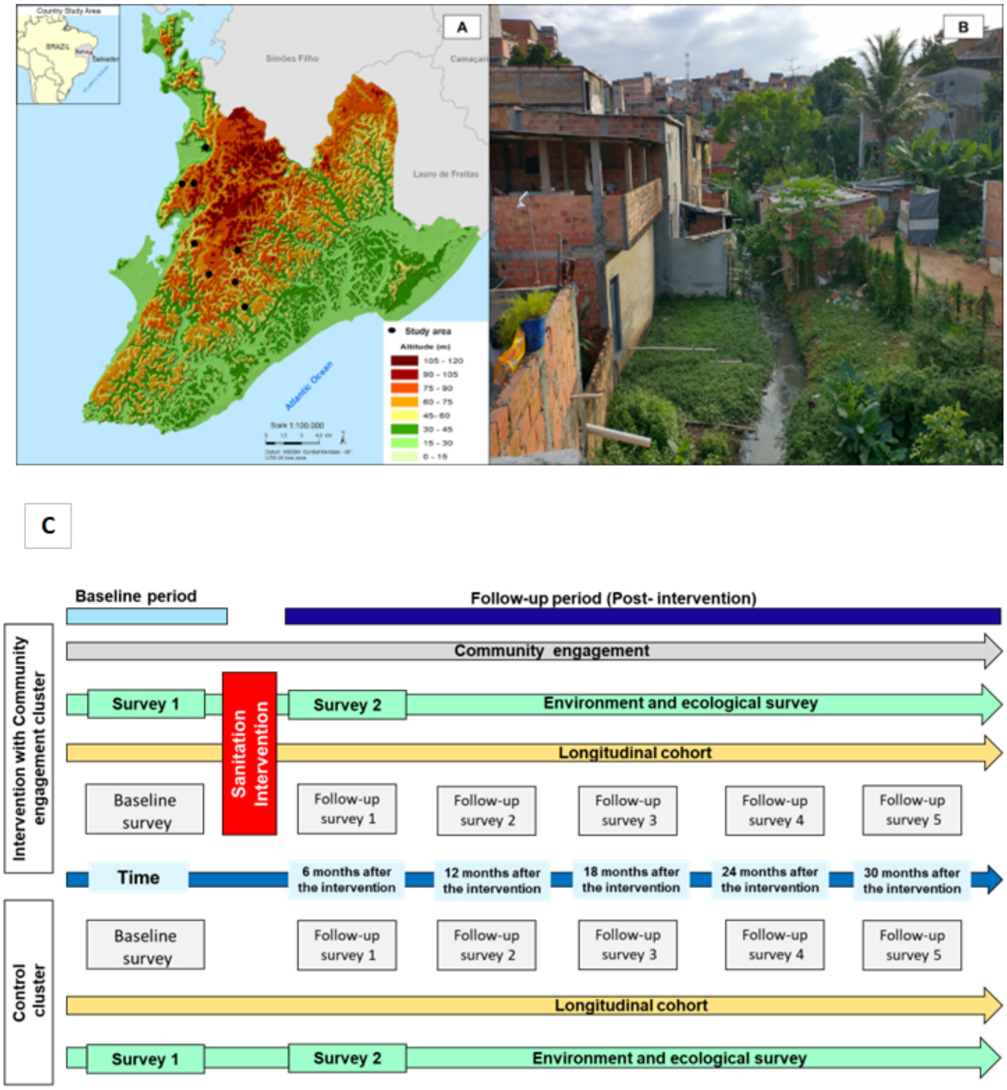

Figure 2- Study clusters in the city of Salvador, Brazil. Altitude and sampling clusters (A). Example of baseline sanitation conditions of the study cluster (B). In (C) Consort flow diagram by intervention and control cluster, data collection times and type of activities.

151x165mm (120 x 120 DPI)

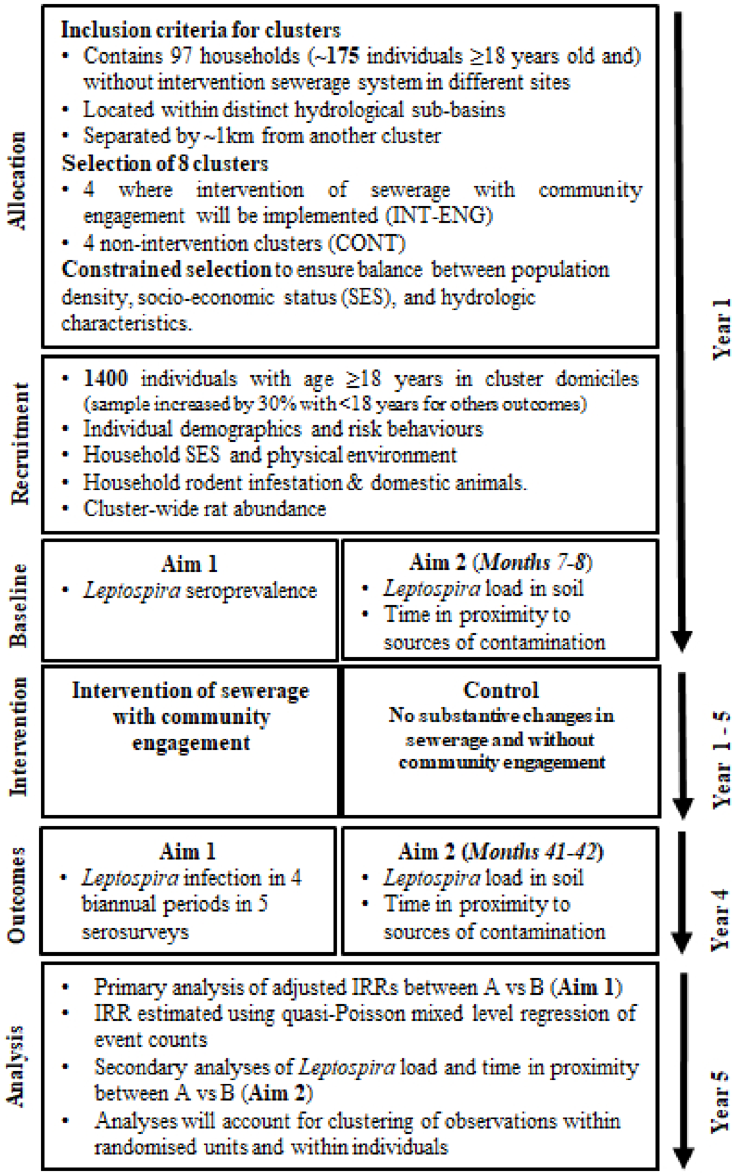

Figure 3- Overview of the study procedures, the timeline for study, and data collection. Note 1: Each cluster of the study will be formed by a cluster with intervention vs. Control. The period of activities for each serological survey will take place at the time described in the figure (Allocation, Recruitment, and Baseline). Note 2: Update years 1 to 5 will be realized after receipt of the water and sanitation company schedule. SES: Socio-economic status.

96x136mm (120 x 120 DPI)

Supplementary material

Supplementary Table 1. Specific age range for each outcome.

Outcome	Age group
Leptospirosis, preventive practice, well-being, perceptions of residents	≥ 18 years
Arboviral diseases	≥ 6 months
Long-term infant development	≥ 6 to ≤ 66 months
Enteric diseases	≥ 6 to ≤ 66 months

For peer review only

Supplementary Table 2. Primary and secondary outcomes baseline in assessed in this study protocol, Salvador, Brazil, 2022

Outcomes of interest	Operational definition
**1. Individual *Leptospira* infection**	
	> *Leptospira* infection defined as a four-fold rise in microscopic agglutination test (MAT) titer or seroconversion (negative to ≥1:50) between samples from consecutive serosurveys.
**2. Individual risk for *Leptospira* exposure**	
	> Walk-in flooding in the neighborhood
	> Walk barefoot outside the household
	> Need to unclog sewerage
	> Perception of vulnerability to leptospirosis
	> Perception of the severity of the leptospirosis disease
	> Perception of the presence of rats near the house
**3. Environmental for *Leptospira* exposure**	
	> Proximity and contact frequency to open sewer, trash, and other exposures to environmental sources
	> Sewage infrastructure at home and in the community
	> Flooding in the community and inside the home
	> Floor and road conditions
	> Accumulation of garbage near the house
	> Presence and concentration of *Leptospira* load in the soil
**4. Sanitation-related knowledge and practice**	
	Consequences of lack or poor sanitation as:
	> Structural problems
	> Major related diseases
	> Institutions responsible for providing sanitation in the community
	> Practice related to sanitation in-home and community
**5. Social Capital**	
	> Self-Assessment of coexistence and trust with neighbors.
	> Perception of neighbors' willingness to help each other
	> Social, leisure, and community support activities practiced
	> Willingness to continue living in the community
**6. Well-being**	
	Well-being will be evaluated through measures such as:
	> Productivity through the assessment of income and employment
	> Quality of health life (SF-12 questionnaire).
	> Food insecurity (EBIA-5 questionnaire)
	> Loss of goods, services, and income due to flooding and landslides
**7. Long-term child development**	
	> Global and specific development in gross and fine motor coordination, communication, problem-solving and personal-social domains (ASQ-3 questionnaire)
	> Performance evaluation in Portuguese and Mathematics tests of Brazilian elementary education (Andres)
	> Anthropometric measures

For peer review only

**8. Enteric diseases**	
	The self-reported gastrointestinal infection will be evaluated through caregiver-reported symptom data in the last 7 days including:<br>> Diarrhoea (≥3 loose or liquid stools in a 24h period)
	> Vomiting
	> Abdominal pain
	> Refusal to eat
	> Hygiene practices
**9. Arboviral diseases**	
	> Accumulation of water in containers after rain
	> Presence of mosquitoes at home
	> Main time of the presence of mosquitoes at home
	> Diagnosis of an arbovirus in the last 6 months

For peer review only

Supplementary Figure 1. Schematic layout of the design of the conventional and simplified sewerage systems.

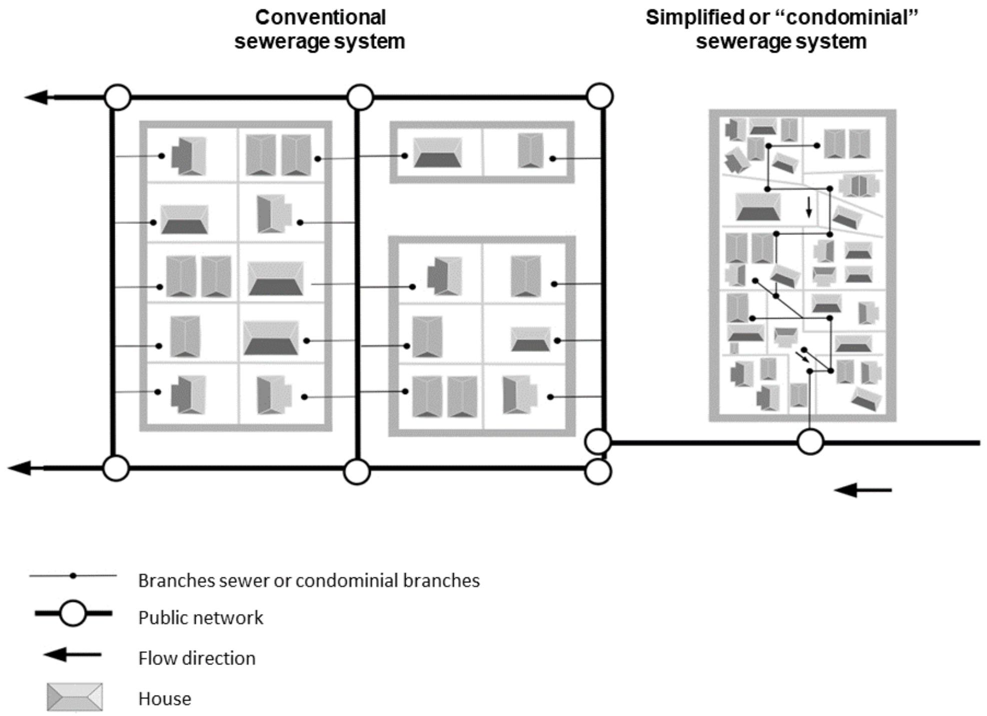

w only

BMJ Open

Simplified sewerage to prevent urban leptospirosis transmission: a cluster non-randomized controlled trial protocol in disadvantaged urban communities of Salvador, Brazil

Journal:	*BMJ Open*
Manuscript ID	bmjopen-2022-065009.R2
Article Type:	Protocol
Date Submitted by the Author:	17-May-2023

Complete List of Authors:	Cremonese, Cleber; Federal University of Bahia,<br>SOUZA, FABIO; Universidade Federal da Bahia<br>Palma, Fabiana ; Federal University of Bahia<br>Sodré, Jonatas Fernandes; Federal University of Bahia<br>Brito, Ricardo; Universidade Federal da Bahia<br>Ribeiro, Priscyla dos Santos ; Federal University of Bahia<br>Santana, Juliet ; Federal University of Bahia<br>Coelho, Rachel Helena; Federal University of Bahia<br>Ticona, Juan P. Aguilar ; Federal University of Bahia<br>Nazaré, Romero ; Fundação Oswaldo Cruz<br>de Oliveira, Daiana ; Federal University of Bahia<br>Silva, Cainã ; Federal University of Bahia<br>Eyre, Max ; Lancaster University<br>Mendes, Vinícius; Universidade Federal da Bahia<br>Knee, Jackie ; London School of Hygiene and Tropical Medicine Faculty of Infectious and Tropical Diseases<br>Ristow, Paula ; Federal University of Bahia<br>Stauber, Christine; Georgia State University<br>López, Yeimi Alexandra; Federal University of Bahia<br>Giorgi, Emanuele; Lancaster University Lancaster Medical School,<br>Diggle, Peter; Lancaster University, Faculty of Health and Medicine; University of Liverpool, Epidemiology and Population Health<br>Reis, Mitermayer Galvão ; Fundação Oswaldo Cruz<br>Cumming, Oliver; London School of Hygiene and Tropical Medicine, Department of Disease Control<br>Ko, Albert; Yale University,<br>Costa, Federico ; Federal University of Bahia

**Primary Subject Heading**:	Global health
Secondary Subject Heading:	Infectious diseases, Public health, Research methods
Keywords:	EPIDEMIOLOGY, Public health < INFECTIOUS DISEASES, BACTERIOLOGY

SCHOLARONE™
Manuscripts

Title: Simplified sewerage to prevent urban leptospirosis transmission: a cluster non-randomized controlled trial protocol in disadvantaged urban communities of Salvador, Brazil

Author affiliations

Cleber Cremonese[1†], Fábio Neves Souza[1,2,3†], Fabiana Almerinda Gonçalves Palma[1,2†], Jonatas Fernandes Araújo Sodré[1], Ricardo Lustosa Brito[1], Priscyla dos Santos Ribeiro[4], Juliet Oliveira Santana[2], Rachel Helena Coelho[5], Juan P. Aguilar Ticona[1,2], Romero Nazaré[2], Daiana de Oliveira[1,2], Cainã Queiroz Silva[1], Max T. Eyre[6], Vinícius de Araújo Mendes[5] ,Jackie Knee[7], Paula Ristow[3], Christine Stauber[8], Yeimi Alexandra Alzate López[1], Emanuele Giorgi[6], Peter John Diggle[6], Mitermayer Galvão dos Reis[2,9], Oliver Cumming[7], Albert I. Ko[2,10] and Federico Costa[1,2,6,10]

[1]Institute of Collective Health, Federal University of Bahia, 40110-040, Salvador, Bahia, Brazil

[2]Instituto Gonçalo Moniz, Fundação Oswaldo Cruz, Ministério da Saúde, Rua Waldemar Falcão, 121, Salvador, Bahia, Brasil

[3]Institute of Biology, Federal University of Bahia, Ondina Campus, 40170-115, Salvador, Bahia, Brazil

[4]Institute of Biological Science, Federal University of Minas Gerais, Belo Horizonte, Brazil

[5]Faculty of Economics, Federal University of Bahia, Dois de Julho, Salvador, 40070-010, Salvador, Bahia, Brazil

[6]Lancaster Medical School, Lancaster University, Lancaster, LA1 4YW, UK

[7]London School of Hygiene & Tropical Medicine, Faculty of Infectious Tropical Diseases, Disease Control Department, United Kingdom

[8]Department of Population Health Sciences, School of Public Health, Georgia State University, Atlanta, GA, USA

For peer review only

[9]Faculty of Medicine of Bahia, Federal University of Bahia, Praça Conselheiro Almeida Couto, s/n - Largo do Terreiro de Jesus, Salvador, BA 40025-010, Salvador, Bahia, Brazil

[10]Department of Epidemiology of Microbial Diseases, School of Public Health, Yale University, 60 College Street, P.O. Box 208034, New Haven, CT, USA

† These authors equally contributed.

Correspondence to: Cleber Cremonese (cleber.cremonese@ufba.br) and Federico Costa (federico.costa@ufba.br)

For peer review only

ABSTRACT

Introduction

Leptospirosis is a globally distributed zoonotic and environmentally mediated disease that has emerged as a major health problem in urban slums in developing countries. Its etiological agent is bacteria of the genus *Leptospira,* which are mainly spread in the urine of infected rodents, especially in an environment where adequate sanitation facilities are lacking, and it is known that open sewers are key transmission sources of the disease. Therefore, we aim to evaluate the effectiveness of a simplified sewerage intervention in reducing the risk of exposure to contaminated environments and *Leptospira* infection and to characterize the transmission mechanisms involved.

Methods and Analysis

This matched quasi-experimental study-design using non-randomized intervention and control clusters was designed to assess the effectiveness of an urban simplified sewerage intervention in the low-income communities of Salvador, Brazil. The intervention consists of household-level piped sewerage connections and community engagement and public involvement activities. A cohort of 1,400 adult participants will be recruited and grouped into eight clusters consisting of four matched intervention-control pairs with approximately 175 individuals in each cluster in baseline. The primary outcome is the seroincidence of *Leptospira* infection assessed through five serological measurements: one pre-intervention (baseline) and four post-intervention. As a secondary outcome, we will assess *Leptospira* load in soil, before and after the intervention. We will also assess *Leptospira* exposures before and after the intervention, through transmission modeling, accounting for residents' movement, contact with flooding, contaminated soil and water, and rat infestation, to examine whether and how routes of exposure for *Leptospira* change following the introduction of sanitation.

Ethics and dissemination

This study protocol has been reviewed and approved by the ethics boards at the Federal University of Bahia and the Brazilian National Research Ethics Committee. Results will be

For peer review only

disseminated through peer-reviewed publications and presentations to implementers, researchers, and participating communities.

Trial registration number: Brazilian Clinical Trials Registry, RCT trial RBR-8cjjpgm.

STRENGTHS AND LIMITATIONS OF THIS STUDY

- The first rigorous controlled intervention study for the effect of sanitation on *Leptospira* transmission in disadvantaged urban communities.
- This is a cluster-based non-randomized controlled trial with intervention clusters receiving a simplified sanitation intervention and matched control clusters no intervention.
- The alliance between the natural and social sciences and the potential that it has to deepen community perceptions about leptospirosis and interventions before and after, as well as involvement in it in some decision-making processes in the improvement of their health, wellbeing, and sanitation.
- The allocation is non-randomized with selection based on intervention criteria determined by the implementer may have diluted intervention effects.
- Intervention will be carried out by the local public water and sanitation company, following their own planning, with no input or interference from the research team in the schedule or implementation.

INTRODUCTION

Globally, leptospirosis is a leading zoonotic disease in terms of morbidity and mortality [1], causing >1 million cases and >50,000 deaths yearly [1–3]. This zoonosis is an environmental and sanitation-related disease [4] characterized by annual epidemics during periods of increased rainfall and flooding [5,6]. Infection occurs frequently following exposure to water and soil that has been contaminated with the urine of infected animals [7,8]. Leptospirosis is an emerging disease in urban areas of most low- and middle-income countries (LMICs) [1], where a significant fraction of the population lives in disadvantaged urban communities (often called slums or *favelas* in Brazil) [9]. In those settings, poverty, precarious housing conditions, overpopulation, and lack of basic sanitation enable a high density of rats (the main urban reservoir hosts [10–13]) and high leptospirosis transmission [14,15]. As the population living in urban disadvantaged communities

For peer review only

will double to 2 billion by 2025 [16] the burden of leptospirosis is expected to increase.

Public health intervention strategies to prevent leptospirosis transmission are essential and must target the infrastructure deficiencies to prevent or diminish human exposure to environmental contamination sources. Governmental interventions focused on improving sanitation are critical for reducing the burden of leptospirosis as well as other diseases, like diarrheal [4,17]. However, these interventions, especially in disadvantaged urban communities, are not a priority or foreseeable soon considering the high cost of providing and maintaining such sanitation infrastructure. Exclusion of the disadvantaged urban communities' health issues in the government's political agenda [18–20], contributes to historical and structural disparities. Brazil, since the eighties, has been recognized as a global leader in the development of cost-effective water and sanitation systems for densely populated urban settings [21,22]. These systems, called simplified or “condominial” water and sewerage systems, are designed to be installed in densely populated, informal communities that are often situated in the kind of hilly terrain, common in Brazilian *favela* communities. The simplified sewerage approach is unique because it redefines the service unit as a community block rather than an individual household. It uses smaller pipes and inspection chambers (or cleanouts) at key junctions to facilitate installation and maintenance [21,22]. The system design reduces costs by decreasing the length of the trenches needed for sewerage lines and by using smaller pipes made from less expensive materials such as PVC (polyvinyl chloride). The installation of this alternative system can cost 50-70% less than a conventional system [21]. The lower costs associated with the installation of simplified systems makes these systems a more feasible alternative to conventional sewer systems in poor, resource-constrained settings. However, the simplified sewerage approach requires intensive community mobilization and engagement [23] as it is residents who take on leadership roles and have responsibility for decision-making.

Health impact evaluations of simplified sewerage approach in preventing disease has previously been limited to diarrheal diseases [24]. The evaluation of such interventions is challenging because of methodological problems such as cost and time of implementation, the requirement of large sample sizes, difficulties with randomization of the intervention allocation, and confounding factors (e.g., rodent infestation and Social-Economic Status). Contact with environments contaminated with sewer overflow or residents' exposure to open sewerage near

For peer review only

the household can influence the degree of individual exposure to *Leptospira* bacteria[14]. Humans exposure to *Leptospira* contamination through poor sanitation is mediated by individual behavior, socio-economic and environmental factors, rainfall, and flooding [4]. Therefore, the mechanisms of *Leptospira* transmission have different but important components of the environmental transmission route, for which their relative contribution is unknown because of confounding factors. Only studies that integrate a rigorous characterization of contamination sources (*Leptospira* load) and residents' movement could separate their effects and understand how source reduction interventions impact those transmission mechanisms. Determining the contribution of each of these mechanisms to *Leptospira* infection would help us to simultaneously understand the relationships and pathways that drive transmission [4]. This information is key to making interventions generalizable to other environmentally transmitted diseases that affect urban disadvantaged populations.

In this protocol, we describe an evaluation of a simplified sewerage intervention and community engagement with a quasi-experimental study-design using non-randomized intervention and control clusters. We aim to assess the longitudinal impact of a simplified sewerage intervention on the incidence and transmission mechanisms of *Leptospira* infection. In addition, we intend to investigate the impact of these interventions on reducing other water and environmental-related transmissible diseases.

Research aims and Hypotheses

We hypothesize that the combination of a simplified sewerage infrastructure and community engagement activities will reduce the risk of *Leptospira* infection among disadvantaged urban communities in Salvador, Brazil. We further hypothesize that this approach will be effective in reducing: 1) frequency of human contact with open sewerage contaminated with pathogenic *Leptospira*; 2) rat infestation; and 3) *Leptospira* load in the soil around the simplified sewerage**.** We aim to evaluate the efficacy of a sewerage intervention on reducing *Leptospira* infection and other health outcomes related to sanitation in disadvantaged urban communities in Brazil. Our specific objectives are to: (1) to evaluate prospectively the effectiveness of a simplified sewerage intervention in reducing the risk of *Leptospira* infection; (2) to determine the mechanisms by which the simplified sewerage reduce direct human contact with environmental pathogen load in urban slums. **Figure 1** provides further details on our hypotheses

For peer review only

concerning the effects of the sanitation intervention on the transmission mechanisms of urban leptospirosis. We conceptualize this protocol as focusing on the sanitation intervention to decrease leptospirosis; however, this study protocol generates rich opportunities to evaluate other health outcomes. Therefore, we additionally aim to investigate the effects of the sewerage intervention on childhood exposure to a range of enteropathogens, and long-term child neurodevelopment and anthropometry. As exploratory outcomes, we will also investigate the effect of the sewerage intervention on the reduction of risk of arbovirus infectious diseases.

METHODS AND ANALYSIS

Overview of study design

This is an intervention trial with a cluster non-randomised controlled trial, with eight clusters (four intervention and four control), including longitudinal serological follow-up of 1,400 adults' residents from disadvantaged urban communities at high risk for leptospirosis transmission in Salvador, Bahia, Brazil. The primary outcome will be *Leptospira* infection. Each pair of intervention and control clusters will be allocated in distinct hydrological sub-basins with distances from 1,000 to 1,500 meters apart. Clusters will be matched on socio-economic status (SES), topographic or hydrologic characteristics, population density, and *Leptospira* seroprevalence. The cluster selection will also consider exposure to the pathogen by selecting houses that are located to similar distances to new sewerage system and control open sewers. The intervention aims at a combination of sanitation (simplified sewerage intervention) and community engagement activities.

Study settings

Salvador is the third largest city in Brazil with a population of 2.9 million residents [25]. Using a surveillance database of active leptospirosis in the study setting between 1996 and 2018, we performed a population-based, adjusted Kernel analysis among geo-localized records of households with leptospirosis cases to identify high risk area corresponding to 8.06% of the total area of the city [15,26]. We selected eight clusters (four intervention and four control) with average size 0.03 Km^2 located in these areas. The intervention clusters will be located in areas where the provision of simplified sewerage and community engagement is planned. Moreover, the choice of

For peer review only

177 the control group is dependent on the implementer's information regarding non-intervention planned in the area for a short-term period of 5 years. Geographic location of intervention and control clusters is detailed in **Figure 2**.

Description of the Sewerage intervention

During 2022 and 2023, the sewerage intervention (simplified sewerage intervention) will be implemented by the Public Water and Sanitation Bahia State Company (*Empresa Baiana de Águas e Saneamento*) in different urban locations in Salvador, Brazil. The expansion of the sanitation coverage in the city has generated an opportunity to assess the longitudinal effectiveness of the intervention on the transmission of sanitation-related diseases, such as leptospirosis. The company is government-owned, with responsibilities for managing, monitoring, and distributing water and sanitation services throughout the state of Bahia. The costs, management, and entire execution of the work will be the responsibility of the water and sanitation company, while the team of researchers will only monitor the development of the work.

This simplified sewerage intervention is characterized by receiving the sewage generated by a cluster of houses instead of individual houses. In conventional sewerage system designs, each household is connected directly to a principal line while in the sewer simplified system households are connected to branch lines within each block [24] **(Supplementary figure 1)**.

The sanitation company will carry out the sewerage network works, which include the installation of a 150 mm primary pipe at the bottom of the residential lot, bordering a watercourse. Household sewerage networks are interconnected on one main line connecting to the main network, thus the intervention is expected to increase sanitation coverage in the study clusters by expanding the collection network. These interventions would exclusively be of a sewerage nature, with no complementary functions such as local drainage. Importantly, the intervention also includes community participation in the action designs and implementation.

Community engagement

Simplified sewerage includes different degrees of social participation. We recognize that the intervention in sanitary sewerage impacts the local environment and the communities' perception is affected by the sanitation interventions. Problems and failures related to sewerage intervention

For peer review only

have been reported as mainly due to the difficulties in the relationship between the technicians who accompany the works and the communities which reduced the community participation to sewerage maintenance [23].

Faced with this complexity related to urban sanitation, the community engagement processes that will be implemented in this study are focused on approaches that would stimulate community mobilization to participate in the research processes, popular health education actions, and in the evaluation of the implementation of sewerage intervention. Popular Health Education (PHE) actions will be oriented toward disease prevention and health promotion of the population. The PHE is a "set of practices targeted at individuals and the community" [27,28], and this study intends to contribute to community empowerment and support for the management of sewerage intervention. The community engagement activities would be implemented in stages and with different stakeholders, including the community leaders, residents, and a multidisciplinary team of researchers including epidemiologists, ecologists, sanitation engineers, anthropologists, environmental microbiologists, geographers, economists, statisticians, etc. The study, therefore, aims to strengthen community participation in the decision-making in the management of sewerage intervention, and also support the involvement of residents in activities research such as (1) Community and institutional mobilization; (2) Collaborative mapping; and (3) Popular health education actions. Moreover, we will perform community ethnography to deepen the understanding of the social and cultural impacts caused before and after the intervention, as well as its environmental impacts and the mobilization processes.

Study population and sample size

To evaluate the effectiveness of the simplified sewerage intervention in reducing the risk of *Leptospira* infection, we plan to recruit an estimated 1,400 participants aged ≥18 years, residing in the selected clusters (approximately 175 adults per cluster in baseline). To evaluate the intervention effect on secondary (sewerage contact, environmental pathogen load, long-term child development) and exploratory (arbovirus infections, diarrheal disease, and enteric infection) outcomes, we plan to increase our sample size by 420 (30%) participants, aged between 6 months and 17 years, totaling 1,840 people. We considered a potential loss to follow-up rate of 15-20% per year. Therefore, by including additional participants, we ensure the maintenance of

For peer review only

an appropriate sample size for the outcomes that will be studied. All participants from both intervention and control clusters will be followed over a period of three years after the sewerage intervention and a baseline prior to the intervention **(Figure 2)**. In the flow diagram, we provide a timeline and trial study phases in detail (**Figure 3)**.

The sample size was estimated using data on *Leptospira* infection from a previous leptospirosis cohort study in Salvador, Brazil [14]. We divided the previous leptospirosis cohort study [14] into areas of similar size to that the clusters used trial and calculated the between-cluster variation. Of 1,840 individuals participating in the previous cohort, 142 (7.9%) experienced leptospiral infection (k/ICC=0.03). Power calculations are based on a cluster-level using effect sizes (% decrease in incidence between intervention and control clusters) 40, 50, and 60%, assuming an infection rate of 0.08 per person-year. All sample size calculations used methods for matched cluster-randomized trials with the study structure of four matched pairs of clusters. Sample size estimates assumed a 12% annual drop-out rate across the three-year study period.

Control selection

Control clusters (CONT) will be selected in locations with an incomplete sewerage sanitation system. Matching criteria for control clusters include: location in a geographic area that has similar environmental (elevation, hydrologic data) and social (income, education, population density) characteristics to the intervention clusters; and at a distance of 1,000 and 1,500 meters from the intervention cluster. Areas that received major infrastructure interventions and sanitation improvements in the last five years will be excluded from the selection frame for control clusters.

Eligibility and enrolment

Before the recruitment of study participants, a census will be carried out within the selected eight clusters to enumerate all residents from households within <40m distance from an open sewer. This would ensure that households and residents living in areas with a higher risk of leptospirosis transmission are included. At each census, the individual data (name, sex, age) and the geo-coordinates a primary household respondent would be collected. As a further step, we will randomly select population-based cohorts with 95 households (175 participants) per cluster. Residents will be eligible for inclusion in the study, if (1) they are 18 years of age or above, (2) have lived in the area for at least six months, (3) sleep in the household for at least 3 nights per

For peer review only

week, and (4) consent to the study procedures and complete an informed consent form. However, residents will be excluded if: (1) they do not meet all of the inclusion criteria; (2) they cannot provide written informed consent to participate in the study, (3) they refuse or are unable to provide demographic information and biological sample; (4) they are with limited mental health problems or other problems that have implications for survey responses or participation in any other phase of research activity.

For secondary outcomes we will include all children and young between 6 months and 17 years of age will be included in the study **(Supplementary table 1)**. We will apply similar inclusion and exclusion criteria as described but excluding children for which their legal guardians do not provide written informed consent for their participation.

Outcomes

Primary outcome measure

The study trial will consist of three specifics phases: (1) baseline; (2) sanitation intervention implementation; (3) follow-up of the residents and evaluation of the sanitation intervention **(Figure 2)**. During the phase (1) a baseline survey will be conducted to characterize the demographics, socio-economic status and *Leptospira* seroprevalence in each cluster. During the phase (3) biannual serosurveys in the cohorts will be conducted to measure the primary outcome, *Leptospira* asymptomatic infection, and estimate the risk of infection during the dry and rainy seasons. The field team will collect blood samples from eligible participants with age ≥18 years. A blood sample (10mL) will be collected from each study participant and examined for *Leptospira* antigen using the microscopic agglutination test diagnostic (MAT) [4]. MAT is the standard test to determine *Leptospira* infection in longitudinal studies and *Leptospira* infection will be defined as a four-fold rise in MAT titer or seroconversion (negative to ≥1:50) between samples from consecutive serosurveys. We will use a panel of six reference strains: representing *L. interrogans* serovars Autumnalis strain Akiyami A, Canicola strain H. Utrecht IV and Icterohaemorrhagiae strain RGA, *L. borgpetersenii* serovar Ballum strain MUS 127, *L. kirschneri* serovar Cynopteri strain 3522C and Grippothyphosa strain Duysterand the two clinical isolates: *L. interrogans* serovar Copenhageni strain Fiocruz L1-130 (isolated locally in 1996) and *L. santarosai* serogroup Shermani strain LV3954 (isolated locally). This procedure had the same performance in

For peer review only - http://bmjopen.bmj.com/site/about/guidelines.xhtml

identifying MAT seroconversion in our prospective studies [4,15] compared with the WHO recommended panel of 19 reference serovars. The screening will be performed with serum dilutions of 1:50-1:100. When agglutination is observed at 1:100, the sample will be titrated to determine the highest titer. As part of quality control procedures, two independent evaluations will be conducted for 8% of the samples and all samples from infected subjects.

Secondary outcomes

Environmental exposure secondary outcomes

Secondary outcomes for environmental effects will include microbiological assays to assess soil contamination with pathogenic *Leptospira*. The presence and concentration of pathogenic *Leptospira* will be evaluated in the eight clusters. Bases on prevalence the contamination soil with *Leptospira* in studies previous and packing density of 0.4 with minimum distances between collection points, a total of 68 soil samples and 15 water sewage samples will be collected in each cluster and transported refrigerated to the laboratory within six hours for processing. Sampling will take place in duplicate, with intervals of seven to nine days between them. After processing, soil and sewage samples will be stored at -80°C until molecular assays can be performed. The presence and concentration of pathogenic *Leptospira* will be evaluated using a qPCR assay (quantitative polymerase chain reaction), targeting the *lipL32* gene. Samples will be considered positive when the two qPCR replicates show amplification before a Cq of 40 [8]. Environmental variables for rainfall, temperature, landscape (remote sensing using drones), flooding, and hydrological features will also be collected during rainy seasons before and at least three months immediately after the sewerage intervention.

Rats Survey

Rat abundance will be examined at each cluster using tracking boards to quantify the level of rat activity, before and after the interventions. The use of tracking boards to quantify rat presence and activity (as rat abundance proxy) will be performed, following previously described, validated, and optimized methods [29]. Tracking boards will be distributed in each cluster for two days in a row and will be scored daily. The overall level of rat presence for each cluster will be estimated based on the percentage of positive tracking boards and the intensity of marks on each board

For peer review only

before and at least three months immediately after the sewerage intervention. We will compare the abundance and activity of rodents with the environmental leptospiral load in the area.

Human movement

Environmental exposure to *Leptospira* (i.e., proximity to open sewer and unpaved roads; housing conditions; and flooding) will be quantified by studying of the individual movement of the participants in each study cluster. Movement evaluations will be performed biannually, before and after the sewerage intervention. We will focus on a sub-cohort of 320 residents living in each of the eight study clusters, divided into four groups: young males (18–34 years), young females (18–34 years), old males (aged ≥35 years), and old females (aged ≥35 years). We will use GPS data loggers that can measure small-scale movement patterns [30,31].

Forty residents in each of the study clusters will be invited to use a GPS data logger IgotU® GT-600 device (Mobile Action Technology, New Taipei City, Taiwan) [32]. Additionally, we will obtain information about movement and behavior through resident diaries and retrospective mapping before and after the intervention. Diaries will provide qualitative information on the number of hours inside/outside the study cluster and the household; contact duration and nature of the contact (i.e., contact duration with and without use of boots) with contaminated sources; and the activity performed (i.e., working, leisure, etc.) for 7 days. Retrospective mapping will provide information on the past 24 hours' pathways traveled by the residents, and contacts with contamination sources from the residents' perspective [31]. To determine participants' compliance with the study protocol using the GPS data logger and diary entry is defined as adherence for blood collection and direct observations will be conducted for participants ≥18 years of age. Non-compliance is defined as non-adherence to all study steps.

Other outcomes

Human health and wellbeing

The data will be collected pre and post-intervention to evaluate the impact on well-being between arms. Other outcomes related to individual and household health and well-being include subjective measures - self-reported general, physical and emotional well-being using the Short-Form Health Survey-12 (SF-12) [33]; perceived living conditions questions; the short version (five

For peer review only

items) of the Brazilian Food Insecurity Scale [34] which measures the perception and experience of hunger over the past 3 months; and objectives measures - productivity through employment and income. We will also evaluate whether there will be a decrease in material and economic loss measures due to floods and landslides -which often occur in the areas without adequate sanitation.

Long-term child development

Enteric pathogens associated with diarrhea can inhibit a child's ability to absorb nutrients, further exacerbating malnutrition and developmental delays. Studies are indicating a growing association between enteric infections and environmental enteropathy, a disorder characterized by reduced intestinal barrier function, abnormal intestinal morphology, and increased inflammation [35,36]. This disorder is thought to stem from unsanitary conditions that lead to repeated exposures to enteric pathogens. Additionally, poor gut health, chronic immune stimulation and malnutrition, are linked to poor neurodevelopment in children, as well as stunting. In poverty-stricken environments, these nutritional and environmental risks can also affect the mother-child behavior patterns, which are critical for normal development, including a balance between time spent with caring their children and sanitation home issues [37].

Child neurodevelopment will be evaluated using the Ages & Stages Questionnaires 3rd edition (ASQ-3) for children aged 6 to 66 months and with the Brazilian National Assessment of School Performance (ANRESC - *Avaliação Nacional do Rendimento Escolar)* for children older than 66 months, in the pre- and post-intervention period. ASQ-3 is a tool used to monitor the child's developmental progress, it includes fine and gross motor, communication, problem-solving, and personal-social domains [38]. ANRESC is part of the Brazilian Basic Education Assessment System, which included the assessment of Math and Language skills [39]. The child development assessment will also include measurements of weight, height, BMI and head circumference. We will use the WHO Child Growth Standards and the results will be summarized using the Z-score [40].

Enteric diseases

For peer review only

To evaluate the impact of the intervention on enteric pathogen exposure among young children and, we will collect stool and/or rectal swab samples from children aged 6 months to 5 years old in the pre-post-intervention periods. We will use a custom-designed TaqMan Array Card (TAC) to analyse samples for the presence of over 30 enteric pathogens, including viruses, bacteria, protozoa, and helminths. The TAC uses microfluidic technology to simultaneously perform 48 individual quantitative polymerase chain reactions per sample and has been previously validated and used for detection enteric pathogens in stool [41–43].

Arboviral diseases

We will evaluate the effectiveness of the sewerage intervention and the changes in landscape characteristics associated with them, in the spatiotemporal distribution of arbovirus (Dengue, Zika, Chikungunya) vector mosquitoes. We will carry out entomological investigations in private (participants' homes) and public areas with the collection of adult and immature mosquitoes by aspiration [44], with the verification of identified mosquitoes breeding sites, to measure their density. We will verify whether there is a spatiotemporal pattern in the presence and density of vector mosquitoes in the sampled regions, to allow us to investigate the possible factors that may be related to changes in the distribution of exposures before, during, and after the intervention process and whether these interventions have modified ecological indices for vector abundance.

Data Collection and management

Trained interviewers will administer specific questionnaires to participants through face-to-face interviews to collect general demographic information, socio-economic data, exposure factors and individual or collective sanitation practices, and knowledge about leptospirosis and other diseases. In the case of children at home, a new questionnaire will be answered by the caregiver. Information will be collected on the child's general health status, development, and medical history and diseases **(Supplementary table 2)**. The questionnaires and all information will be confidential and coded using individually identifiable numeric identifiers. Every effort will be made to protect the privacy of the study participants and all data collected will be treated as confidential.

All questioners and forms described in this protocol will be included in the Research Electronic Data Capture (REDCap) database. The REDCap web version as well as its mobile application

For peer review only

will be used to record all data using mobile devices. A data management staff will keep track of the information in real time.

Participants and public involvement

All study subjects will be involved in the trial, answering questionnaires, providing biological samples, and involved in engagement activities as previously described in the section on community engagement. The sanitation intervention will be conducted by a governmental agency, and the implementation of this intervention involves dialogue with the residents of the communities. However, the study design and conduct of the trial of this intervention study will be taking place independently of public involvement. After each follow-up completion, research findings will be shared and disseminated in a group's meetings or visit households with key stakeholders.

Statistical analysis

Descriptive statistics will be employed to report the demographic, social, economic, or environmental characteristics between the study clusters (intervention and control clusters). For the primary outcome, we will apply quasi-Poisson regression to estimate the incidence rate ratio (IRR) of *Leptospira* infection. We will also evaluate the effect of the intervention on *Leptospira* infection risk using mixed-effects logistic regression models to account for clustering of the observations within study units and across repeated measures for individuals. We will model the overall effect of the intervention on infection risk while controlling for baseline sewerage and flooding exposure and potential confounding variables, such as age, sex, indicators of the socio-economic level, and rainfall intensity during an annual period, which are presumably unrelated to the intervention **(Figure 1)**.

For the secondary outcome, we will conduct environmental analyses of *Leptospira* load between intervention and control clusters to compare environmental risk. To do this, we will model two leptospiral load outcomes, prevalence (binomial) and concentration (log-transformed), using generalized linear models with random effects to account for clustering within study units. The association between environmental contamination and hypothesized predictors of risk (rat abundance, human movement, and ecological data) will also be explored in the model **(Figure 1)**.

For peer review only

Limitations and bias

The allocation is non-randomised with selection based on intervention criteria determined by the implementer so the risk of residual confounding cannot be excluded. The choice of the control group is also dependent on the implementer's information about the non-intervention in the area in the short term (5 years-period). To limit the potential for bias arising from our non-randomised design, we will select control group with as similar as possible socio-economic and environmental characteristics to those of their matched intervention clusters.

We will perform the impact calculation through the double differentiation of the averages of the results found between the intervention and control clusters before and after the sewerage intervention. The mitigation of bias will be carried out through the control of fixed effects of time and location between different intervention and control clusters over the years of evaluation.

ETHICS AND DISSEMINATION

This project was evaluated and approved by a local ethics committee (CEP/ISC/UFBA) under number CAEE 32361820.7.0000.5030, and a national research ethics committee (CONEP) linked to the Brazilian Ministry of the Health under approval number 4.235.251. The trial study protocol was registered with the Brazilian Clinical Trials Registry, RCT trial RBR-8cjjpgm (https://ensaiosclinicos.gov.br/rg/RBR-8cjjpgm). All participants involved in the study will provide informed consent before any study activity, and the community leaders, previously identified by the team, will provide verbal consent before enrollment of the community in the trial. Participants aged 12 and 17 will sign an assent form, in addition to a participation consent form completed through their parents or legal guardians. Children under the age of 12 will have their consent signed by their parents or legal guardians only. The results of the trial will be presented at local and international meetings and submitted to peer-reviewed journals for publication. Results will also be shared directly with the participating communities. All individual information participants will be kept and published anonymously.

Authors' contributions

FC, CC, MGR, AK, YAL, PD, CS and OC contributed with project's main conceptual ideas; FC, ME, EG, FNS, FAP and CC contributed to study design and statistical analysis plan; FNS, DO,

For peer review only - http://bmjopen.bmj.com/site/about/guidelines.xhtml

FAP, CS, JFA, PSR, JOS, RHC, DS, RN, RLB, VAM, JPAT, JK, PR, FC, and CC developed field and laboratory protocols and implementation; FNS, FAP, CC, RHC, and FC developed data collection tools; FNS, FAP, JFA, RHC, PR, CC, and FC contributed to drafting the main manuscript. All authors contributed to editing and revising the manuscript.

Funding statement

This work was supported by Wellcome Trust grant number 102330/Z/13/Z. All project costs are jointly funded by Wellcome Trust and the Department of Health and Social Assistance (DHSC), through the National Institute for Health Research (NIHR), using the UK Official Development Assistance Fund (ODA).

Competing interest statement

Not applicable

Data sharing statement

The data collected with personal identifying data redacted will be available encoded for use by principal investigators on request following the publication of trial results. The funders have no role in the study design, data collection, and analysis, decision to publish, or preparation of manuscripts.

Acknowledgments

The authors gratefully acknowledge critical input on the study protocol and all the study participants. We acknowledge the partner institutions for their collaboration and dedication. We thank Patricia Lustosa, and Mayara Carvalho for their help with the review and discussion of this protocol, Ana Maria Silva and George G. Machado for help with supplementary figure, and Hammed O. Mogaji for his help with manuscript proofing.

REFERENCES

1. Costa F, Hagan JE, Calcagno J, Kane M, Torgerson P, Martinez-Silveira MS, et al. Global Morbidity and Mortality of Leptospirosis: A Systematic Review. PLoS Negl Trop Dis. 2015;9: e0003898.

For peer review only

2. Ko AI, Goarant C, Picardeau M. Leptospira: the dawn of the molecular genetics era for an emerging zoonotic pathogen. Nat Rev Microbiol. 2009;7: 736–747.

3. Bharti AR, Nally JE, Ricaldi JN, Matthias MA, Diaz MM, Lovett MA, et al. Leptospirosis: a zoonotic disease of global importance. Lancet Infect Dis. 2003;3: 757–771.

4. Khalil H, Santana R, de Oliveira D, Palma F, Lustosa R, Eyre MT, et al. Poverty, sanitation, and Leptospira transmission pathways in residents from four Brazilian slums. PLoS Negl Trop Dis. 2021;15: e0009256.

5. Ko AI, M GR, Cm RD, Johnson WD, Riley LW. Urban epidemic of severe leptospirosis in Brazil. Salvador Leptospirosis Study Group. Lancet. 1999;354. doi:10.1016/s0140-6736(99)80012-9

6. Sarkar U, Nascimento SF, Barbosa R, Martins R, Nuevo H, Kalofonos I, et al. Population-based case-control investigation of risk factors for leptospirosis during an urban epidemic. Am J Trop Med Hyg. 2002;66. doi:10.4269/ajtmh.2002.66.605

7. Bharti AR, Nally JE, Ricaldi JN, Matthias MA, Diaz MM, Lovett MA, et al. Leptospirosis: a zoonotic disease of global importance. Lancet Infect Dis. 2003;3: 757–771.

8. Casanovas-Massana A, Pedra GG, Wunder EA Jr, Diggle PJ, Begon M, Ko AI. Quantification of Leptospira interrogans Survival in Soil and Water Microcosms. Appl Environ Microbiol. 2018;84. doi:10.1128/AEM.00507-18

9. Programme UNHS, United Nations Human Settlements Programme. World Cities Report 2020. World Cities Report. 2020. doi:10.18356/27bc31a5-en

10. Minter A, Diggle PJ, Costa F, Childs J, Ko AI, Begon M. Evidence of multiple intraspecific transmission routes for Leptospira acquisition in Norway rats (Rattus norvegicus). Epidemiol Infect. 2017;145: 3438–3448.

11. Panti-May JA, Carvalho-Pereira TSA, Serrano S, Pedra GG, Taylor J, Pertile AC, et al. A Two-Year Ecological Study of Norway Rats (Rattus norvegicus) in a Brazilian Urban Slum. PLoS One. 2016;11: e0152511.

12. Costa F, Wunder EA Jr, De Oliveira D, Bisht V, Rodrigues G, Reis MG, et al. Patterns in Leptospira Shedding in Norway Rats (Rattus norvegicus) from Brazilian Slum Communities at High Risk of Disease Transmission. PLoS Negl Trop Dis. 2015;9: e0003819.

13. Costa F, Porter FH, Rodrigues G, Farias H, de Faria MT, Wunder EA, et al. Infections by Leptospira interrogans, Seoul virus, and Bartonella spp. among Norway rats (Rattus norvegicus) from the urban slum environment in Brazil. Vector Borne Zoonotic Dis. 2014;14: 33–40.

14. Hagan JE, Moraga P, Costa F, Capian N, Ribeiro GS, Wunder EA Jr, et al. Spatiotemporal Determinants of Urban Leptospirosis Transmission: Four-Year Prospective Cohort Study of Slum Residents in Brazil. PLoS Negl Trop Dis. 2016;10: e0004275.

15. Reis RB, Ribeiro GS, Felzemburgh RDM, Santana FS, Mohr S, Melendez AXTO, et al. Impact of environment and social gradient on Leptospira infection in urban slums. PLoS Negl Trop Dis. 2008;2: e228.

16. United Nations Human Settlements Programme. The Challenge of Slums: Global Report on Human Settlements, 2003. UN-HABITAT; 2003.

17. Barreto ML, Genser B, Strina A, Teixeira MG, Assis AMO, Rego RF, et al. Effect of city-wide sanitation programme on reduction in rate of childhood diarrhoea in northeast Brazil: assessment by two cohort studies. Lancet. 2007;370: 1622–1628.

18. Bolay J-C. Slums and Urban Development: Questions on Society and Globalisation. The European Journal of Development Research. 2006;18: 284–298.

19. Staff UNHSP, Programme UNHS. The challenge of slums: global report on human settlements, 2003. UN-HABITAT; 2003.

20. IBGE – Instituto Brasileiro de Geografia e Estatística. In: Censo Demográfico 2020 [Internet]. [cited 23 Dec 2021]. Available: https://www.ibge.gov.br/

21. Melo JC. The experience of condominial water and sewerage systems in Brazil: case studies from Brasilia, Salvador and Parauebas. The World Bank; 2005.

22. Melo JC. Sistema Condominial: uma resposta ao desafio da universalização do saneamento. Ministério das Cidades. 2008.

23. Nance E, Ortolano L. Community Participation in Urban Sanitation: Experiences in Northeastern Brazil. Journal of Planning Education and Research. 2007;26: 284–300.

24. Hubbard B, Sarisky J, Gelting R, Baffigo V, Seminario R, Centurion C. A community demand-driven approach toward sustainable water and sanitation infrastructure development. Int J Hyg Environ Health. 2011;214: 326–334.

25. IBGE. Estimated Population 2017. Cities, Bahía, Salvador. [cited 11 Apr 2022]. Available: https://cidades.ibge.gov.br/brasil/ba/salvador/panorama

26. Srihera R, Stute W. Kernel adjusted density estimation. Stat Probab Lett. 2011;81: 571–579.

27. Seabra CAM, Xavier SPL, Sampaio YPC, de Oliveira MF, da Silva Quirino G, de Fátima Antero Sousa Machado M. Health education as a strategy for the promotion of the health of the elderly: an integrative review. Revista Brasileira de Geriatria e Gerontologia. 2019. doi:10.1590/1981-22562019022.190022

28. Falkenberg MB, Mendes T de PL, Moraes EP de, Souza EM de. Educação em saúde e educação na saúde: conceitos e implicações para a saúde coletiva. Ciênc saúde coletiva. 2014;19: 847–852.

29. Hacker KP, Minter A, Begon M, Diggle PJ, Serrano S, Reis MG, et al. A Comparative Assessment of Track Plates to Quantify Fine Scale Variations in the Relative Abundance of Norway Rats in Urban Slums. Urban Ecosyst. 2016;19: 561–575.

30. Vazquez-Prokopec GM, Stoddard ST, Paz-Soldan V, Morrison AC, Elder JP, Kochel TJ, et al. Usefulness of commercially available GPS data-loggers for tracking human movement and exposure to dengue virus. International Journal of Health Geographics. 2009. p. 68. doi:10.1186/1476-072x-8-68

31. Owers KA, Odetunde J, de Matos RB, Sacramento G, Carvalho M, Nery N Jr, et al. Fine-scale GPS tracking to quantify human movement patterns and exposure to leptospires in the urban slum environment. PLoS Negl Trop Dis. 2018;12: e0006752.

32. Morris G, Conner LM. Assessment of accuracy, fix success rate, and use of estimated horizontal position error (EHPE) to filter inaccurate data collected by a common commercially available GPS logger. PLoS One. 2017;12: e0189020.

33. Ware J Jr, Kosinski M, Keller SD. A 12-Item Short-Form Health Survey: construction of scales and preliminary tests of reliability and validity. Med Care. 1996;34: 220–233.

34. Santos LPD, Lindemann IL, Motta JVDS, Mintem G, Bender E, Gigante DP. Proposal of a short-form version of the Brazilian food insecurity scale. Rev Saude Publica. 2014;48: 783–789.

35. Donowitz JR, Drew J, Taniuchi M, Platts-Mills JA, Alam M, Ferdous T, et al. Diarrheal Pathogens Associated With Growth and Neurodevelopment. Clin Infect Dis. 2021;73: e683.

36. Website. Available: https://nyaspubs.onlinelibrary.wiley.com/doi/10.1111/nyas.12330

For peer review only - http://bmjopen.bmj.com/site/about/guidelines.xhtml

37. George CM, Perin J, Parvin T, Bhuyian MSI, Thomas ED, Monira S, et al. Diarrhea Prevalence and Child Growth Faltering Are Associated with Subsequent Adverse Child Developmental Outcomes in Bangladesh (CHoBI7 Program). Am J Trop Med Hyg. 2022;106: 233.

38. Filgueiras A, Pires P, Maissonette S, Landeira-Fernandez J. Psychometric properties of the Brazilian-adapted version of the Ages and Stages Questionnaire in public child daycare centers. Early Hum Dev. 2013;89: 561–576.

39. Brasil M, da Aneb IM. da Anresc 2015. Brasília: Inep. 2017.

40. de Onis M, Garza C, Victora CG, Onyango AW, Frongillo EA, Martines J. The WHO Multicentre Growth Reference Study: planning, study design, and methodology. Food Nutr Bull. 2004;25: S15–26.

41. Platts-Mills JA, Liu J, Rogawski ET, Kabir F, Lertsethtakarn P, Siguas M, et al. Use of quantitative molecular diagnostic methods to assess the aetiology, burden, and clinical characteristics of diarrhoea in children in low-resource settings: a reanalysis of the MAL-ED cohort study. The Lancet Global Health. 2018;6: e1309–e1318.

42. Liu J, Platts-Mills JA, Juma J, Kabir F, Nkeze J, Okoi C, et al. Use of quantitative molecular diagnostic methods to identify causes of diarrhoea in children: a reanalysis of the GEMS case-control study. Lancet. 2016;388: 1291–1301.

43. Liu J, Gratz J, Amour C, Nshama R, Walongo T, Maro A, et al. Optimization of Quantitative PCR Methods for Enteropathogen Detection. PLoS One. 2016;11: e0158199.

44. Vazquez-Prokopec GM, Galvin WA. A new, cost-effective, battery-powered aspirator for adult mosquito collections. Journal of medical. 2009. Available: https://academic.oup.com/jme/article-abstract/46/6/1256/921254

For peer review only

FIGURES

Figure 1- Rationale, hypothesis, and outcomes assessed in the study. We hypothesize that sanitation intervention will improve the environmental conditions and change the behavior of humans, resulting in both direct and indirect impacts on the incidence of leptospirosis and other diseases human and well-being. * HoH- Household-level

Figure 2- Study clusters in the city of Salvador, Brazil. Altitude and sampling clusters (**A**). Example of baseline sanitation conditions of the study cluster (**B). I**n (**C)** Consort flow diagram by intervention and control cluster, data collection times and type of activities.

Figure 3- Overview of the study procedures, the timeline for study, and data collection. **Note 1**: Each cluster of the study will be formed by a cluster with intervention vs. Control. The period of activities for each serological survey will take place at the time described in the figure (Allocation, Recruitment, and Baseline). **Note 2**: Update years 1 to 5 will be realized after receipt of the water and sanitation company schedule. SES: Socio-economic status.

For peer review only

For peer review only - http://bmjopen.bmj.com/site/about/guidelines.xhtml

For peer review only

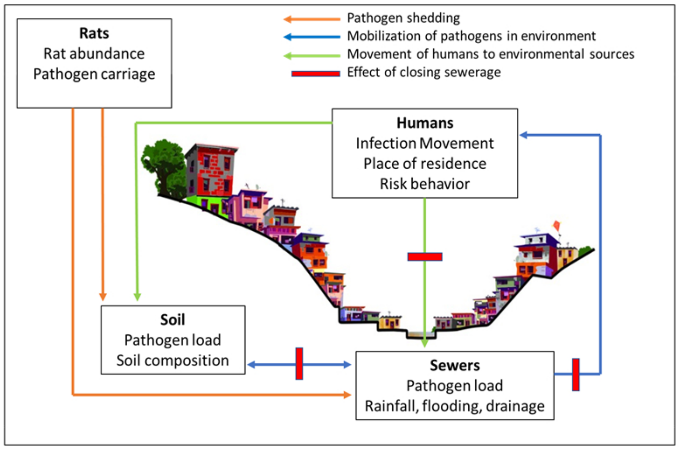

Measurements	
**Ascertainment**	**Measure**
**Cluster-level**	
Nat. Meteorol. Serv	Weather
Remote sensing survey	Land classification
GIS survey	Topology
	Hydrology, flood-risk
Environmental surveys	Open drainage
	Open refuse deposits
Intervention Company Database	Sewerage connections
Tracking board surveys	Rat abundance
Soil *lip*L32 qPCR survey	*Leptospira* DNA load
**Household-level**	
Interviews of HoH	Socio-economic Status
Environmental surveys	Informal sewerage
	Sewage line condition
Rodent infestation survey	Infestation score
Census	Domestic animals
**Individual-level**	
Engagement Communitarian	Community participation level
Interviews	Demographics
	Risk behaviours
	Time in cluster
Serosurveys	*Leptospira* infection
GPS tracking survey	Time in proximity to sources of contamination

Figure 1- Rationale, hypothesis, and outcomes assessed in the study. We hypothesize that sanitation intervention will improve the environmental conditions and change the behavior of humans, resulting in both direct and indirect impacts on the incidence of leptospirosis and other diseases human and well-being.

242x108mm (120 x 120 DPI)

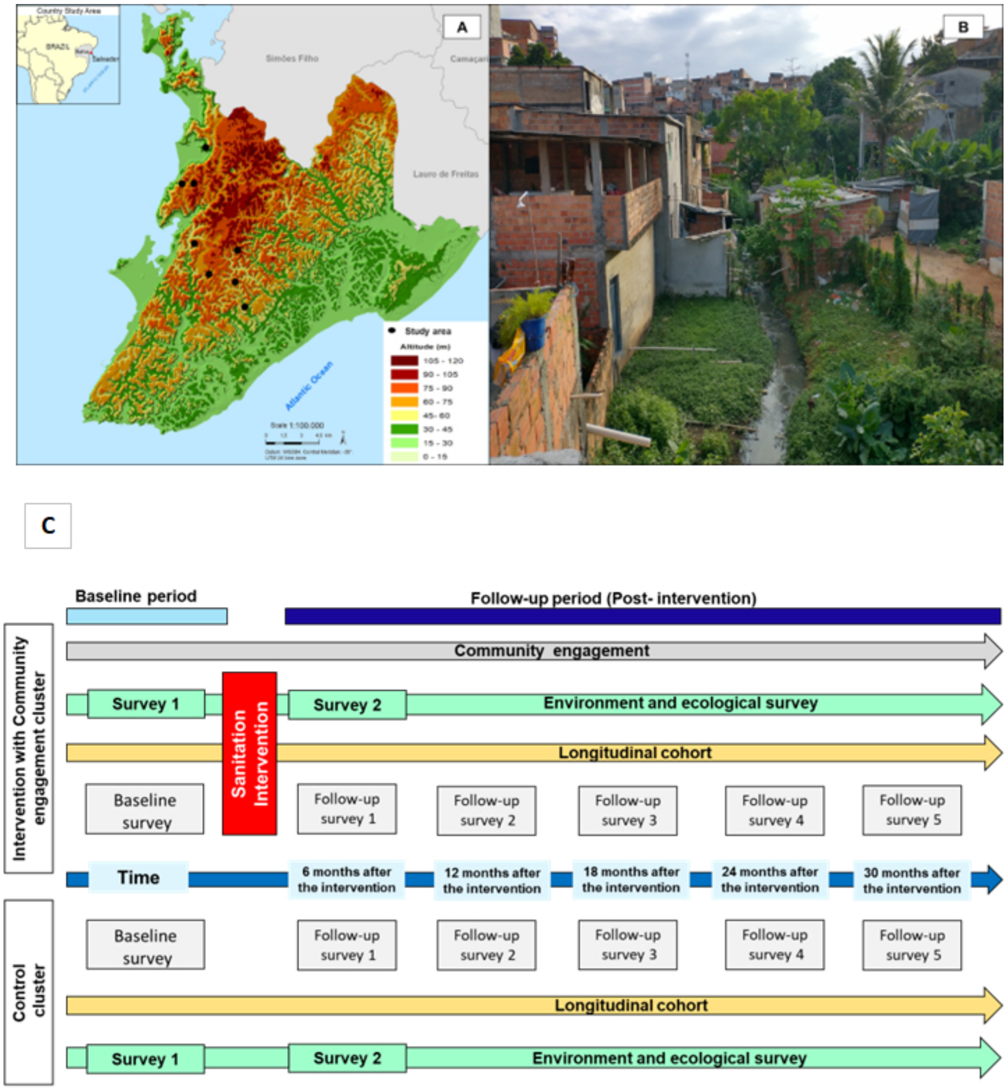

Figure 2- Study clusters in the city of Salvador, Brazil. Altitude and sampling clusters (A). Example of baseline sanitation conditions of the study cluster (B). In (C) Consort flow diagram by intervention and control cluster, data collection times and type of activities.

151x165mm (120 x 120 DPI)

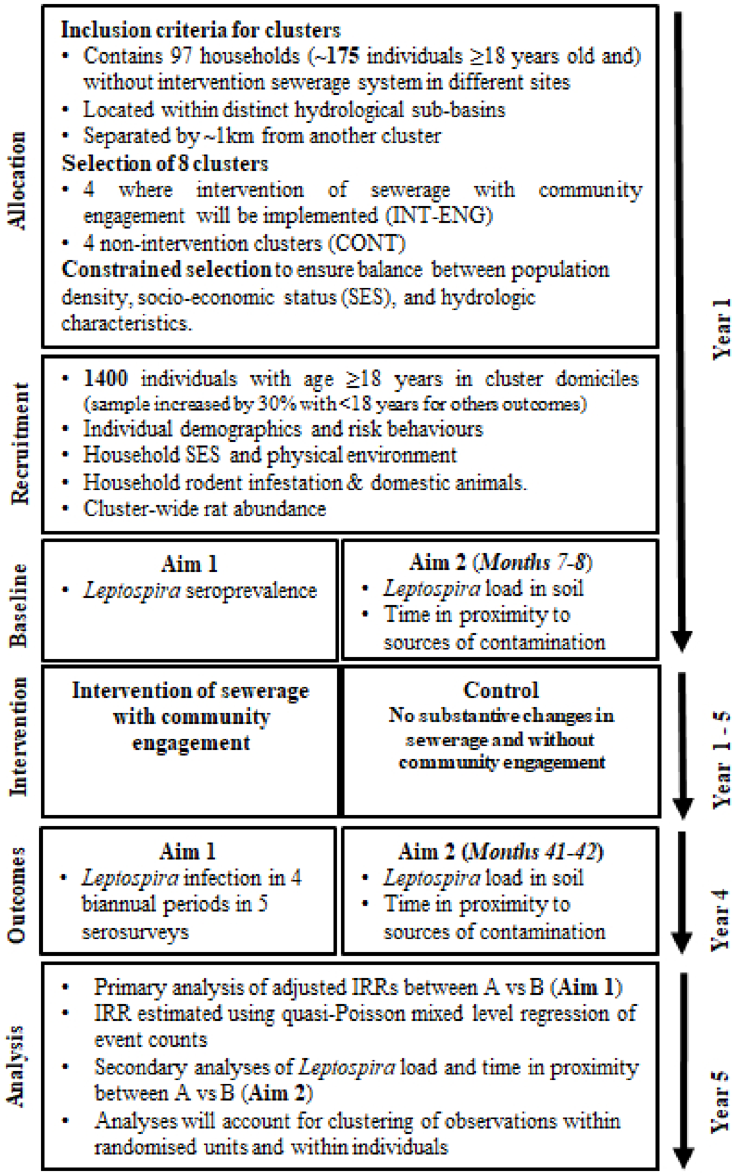

Figure 3- Overview of the study procedures, the timeline for study, and data collection. Note 1: Each cluster of the study will be formed by a cluster with intervention vs. Control. The period of activities for each serological survey will take place at the time described in the figure (Allocation, Recruitment, and Baseline). Note 2: Update years 1 to 5 will be realized after receipt of the water and sanitation company schedule. SES: Socio-economic status.

96x136mm (120 x 120 DPI)

Supplementary material

Supplementary Table 1. Specific age range for each outcome.

Outcome	Age group
Leptospirosis, preventive practice, well-being, perceptions of residents	≥ 18 years
Arboviral diseases	≥ 6 months
Long-term infant development	≥ 6 to ≤ 66 months
Enteric diseases	≥ 6 to ≤ 66 months

For peer review only

Supplementary Table 2. Primary and secondary outcomes baseline in assessed in this study protocol, Salvador, Brazil, 2022

Outcomes of interest	Operational definition
**1. Individual *Leptospira* infection**	
	> *Leptospira* infection defined as a four-fold rise in microscopic agglutination test (MAT) titer or seroconversion (negative to ≥1:50) between samples from consecutive serosurveys.
**2. Individual risk for *Leptospira* exposure**	
	> Walk-in flooding in the neighborhood
	> Walk barefoot outside the household
	> Need to unclog sewerage
	> Perception of vulnerability to leptospirosis
	> Perception of the severity of the leptospirosis disease
	> Perception of the presence of rats near the house
**3. Environmental for *Leptospira* exposure**	
	> Proximity and contact frequency to open sewer, trash, and other exposures to environmental sources
	> Sewage infrastructure at home and in the community
	> Flooding in the community and inside the home
	> Floor and road conditions
	> Accumulation of garbage near the house
	> Presence and concentration of *Leptospira* load in the soil
**4. Sanitation-related knowledge and practice**	
	Consequences of lack or poor sanitation as:
	> Structural problems
	> Major related diseases
	> Institutions responsible for providing sanitation in the community
	> Practice related to sanitation in-home and community
**5. Social Capital**	
	> Self-Assessment of coexistence and trust with neighbors.
	> Perception of neighbors' willingness to help each other
	> Social, leisure, and community support activities practiced
	> Willingness to continue living in the community
**6. Well-being**	
	Well-being will be evaluated through measures such as:
	> Productivity through the assessment of income and employment
	> Quality of health life (SF-12 questionnaire).
	> Food insecurity (EBIA-5 questionnaire)
	> Loss of goods, services, and income due to flooding and landslides
**7. Long-term child development**	
	> Global and specific development in gross and fine motor coordination, communication, problem-solving and personal-social domains (ASQ-3 questionnaire)
	> Performance evaluation in Portuguese and Mathematics tests of Brazilian elementary education (Andres)
	> Anthropometric measures

For peer review only

**8. Enteric diseases**	
	The self-reported gastrointestinal infection will be evaluated through caregiver-reported symptom data in the last 7 days including:<br>> Diarrhoea (≥3 loose or liquid stools in a 24h period)<br>> Vomiting<br>> Abdominal pain<br>> Refusal to eat<br>> Hygiene practices
**9. Arboviral diseases**	
	> Accumulation of water in containers after rain<br>> Presence of mosquitoes at home<br>> Main time of the presence of mosquitoes at home<br>> Diagnosis of an arbovirus in the last 6 months

For peer review only

Supplementary Figure 1. Schematic layout of the design of the conventional and simplified sewerage systems.

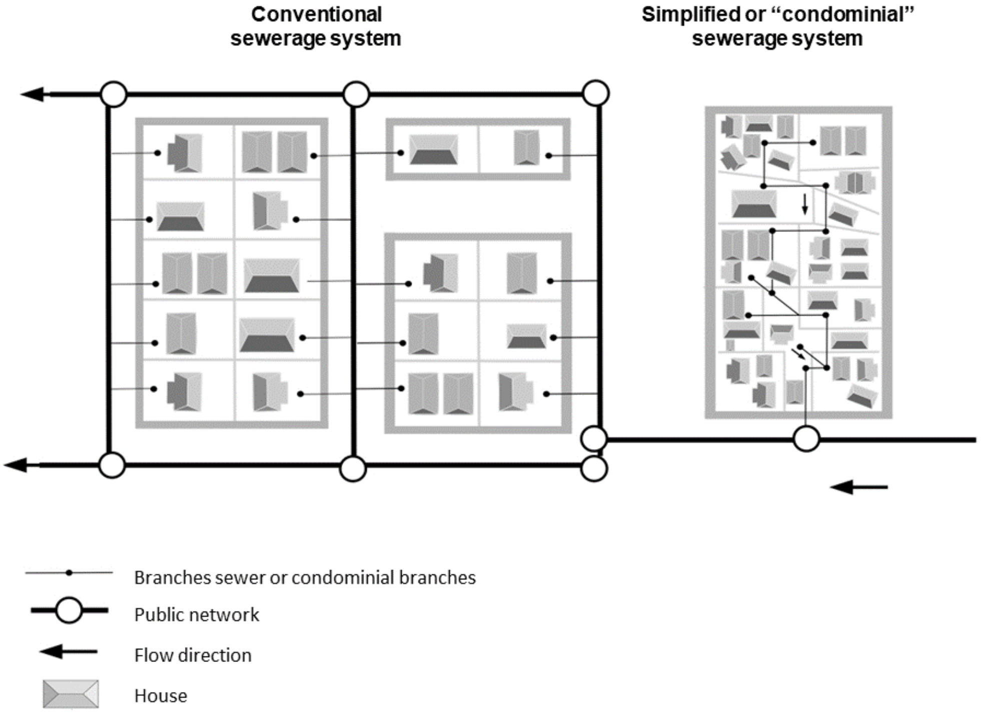

w only
